# Platelet-activating factor receptor antagonists of natural origin for acute ischemic stroke: a systematic review of current evidence

**DOI:** 10.3389/fphar.2022.933140

**Published:** 2022-08-31

**Authors:** Tingting Li, Xuebin Zhang, Ping Jiang, Dandan Zhang, Luda Feng, Xinxing Lai, Mingzhen Qin, Yufei Wei, Chi Zhang, Ying Gao

**Affiliations:** ^1^ Dongzhimen Hospital, Beijing University of Chinese Medicine, Beijing, China; ^2^ Institute for Brain Disorders, Beijing University of Chinese Medicine, Beijing, China; ^3^ Department of Internal Neurology, First Affiliated Hospital, Guangxi University of Chinese Medicine, Nanning, China

**Keywords:** acute ischemic stroke, platelet activating factor receptor antagonists, systematic review, randomized controlled trial, efficacy, safety

## Abstract

**Background:** Acute ischemic stroke (AIS) is a common cause of death and long-term disability worldwide. Recent trials of platelet-activating factor receptor antagonists (PAFRA) appeared to indicate that they could play a neuroprotective role in the treatment of AIS; therefore, we conducted a systematic literature review to evaluate the clinical efficacy and safety of PAFRA in patients with AIS.

**Methods:** A systematic literature search was performed in seven electronic databases from inception to 11 March 2022. All randomized controlled trials (RCTs) in which patients were treated with PAFRA strategies within 7 days of stroke onset were included. Modified Rankin Scale (mRS) was selected as the primary outcome of this systematic review. The methodological quality of included studies was assessed based on the Cochrane Collaborations tool. The review protocol was previously registered (PROSPERO CRD42020182075).

**Results:** Fifteen RCTs comprising a total of 3,907 participants were included in this study. The PAFRA-related compounds included natural preparations of terpenoids, flavonoids, and saponins, namely, ginkgo endoterpene diester meglumine (GEDM, seven RCTs), *ginkgo biloba* dropping pill (GBDP, one RCT), ginkgolide injection (GDI, four RCTs), hesperidin (HES, one RCT), ginsenoside Rd injection (GSRI, one RCT), and hydroxysafflor yellow A (HSYA, one RCT). All studies were conducted in China between 2017 and 2021, employing a two-arm parallel design with sample sizes ranging from 40 to 1,113. Eight studies (53.3%) provided no information on their method of randomization, and only two studies (13.3%) utilized the double-blind design. Treatment was associated with improved clinical outcomes for **(1)** GEDM, GDI, and GBDP in patients treated with conventional treatment (CM) [GEDM + CM for AIS on mRS: MD_mRS_ = −0.42, 95% CI (−0.47, −0.37), five trials, *p* < 0.00001; GEDM + CM for AIS on NIHSS: MD_NIHSS_ = −1.02, 95% CI (−1.51, −0.52), four trials, *p* < 0.0001]; **(2)** GEDM and GDI in patients treated with neuroprotective agent (NPA) [GEDM + NPA + CM for AIS on mRS: MD_mRS_ = −0.40, 95% CI (−0.54, −0.26), *p* < 0.00001; GEDM + NPA + CM for AIS on NIHSS: MD_NIHSS_ = −3.93, 95%CI (−7.72, −0.14), *p* = 0.04]; **(3)** GBDP in patients treated with CM; **(4)** GDI and GSRI in patients treated with IV rt-PA therapy (IVT); and **(5)** HSYA in patients compared with Dengzhan Xixin injection (DZXXI). No access to improved clinical outcome was associated with HES in patients treated with IVT. Seven RCTs reported adverse events (AEs) but found that taking PAFRA-related preparations was not associated with an increased incidence of AEs.

**Conclusions:** This systematic review not only makes an important contribution to the existing body of current evidence but also lays a well-conducted basis for providing opinions and recommendation on the evaluation of PAFRA-based medicine, which could also highlight the need for well-designed clinical trials of PAFRA for AIS to increase the quality of available evidence. Further research is required, using standardized functional outcome measures for AIS, adequate blinding and suitable comparator groups reflecting current best practice.

## 1 Introduction

Stroke is the second leading cause of death worldwide (GBD 2016 Stroke Collaborators, 2019) and is characterized by high rates of incidence, disability, recurrence, and mortality. This disease imposes an enormous health and economic burden on a nation. Acute ischemic stroke (AIS), as a major pathological stroke type, is defined as the sudden loss of blood flow to an area of the brain with a resultant loss of neurological function. The currently available therapies for AIS are based on vascular recanalization strategies including intravenous thrombolysis, endovascular interventions, and antiplatelet, anticoagulation, and other antithrombotic treatments to improve cerebral circulation (Hacke et al., 2008; Mendelson and Prabhakaran, 2021). Regrettably, only a minority of patients receive effective reperfusion therapy in a timely manner due to the low recanalization rate, short therapeutic time window, and numerous contraindications. In addition, dual antiplatelet treatment (DAPT) has become the mainstay in the treatment of AIS (Johnston et al., 2018). However, antiplatelet therapy still has some deficiencies such as individual differences in the efficacy of clopidogrel due to genetic variations in the cytochrome P450 (CYP) gene or adverse drug reactions including respiratory, gastrointestinal, bleeding, and allergic events (Mega and Simon, 2015; Diener and Hankey, 2020). The pathogenesis of stroke is complex, involving not only ischemic tissue necrosis but also post-stroke immune abnormalities, ischemia–reperfusion injury, and other overlapping injury mechanisms, which require comprehensive therapeutic measures such as the use of anti-inflammatory and neuroprotective agents (Chamorro et al., 2016). There has been limited progress in understanding the pathogenesis of stroke, and searching for novel therapeutic approaches has particular clinical implications that become apparent with an updated review of the current body of evidence for AIS.

Platelet-activating factor (PAF) was discovered by the French immunologist Jacques Benveniste in the early 1970s (Benveniste, 1974). The name, PAF, originated from the platelet-aggregating function, and a wide range of physiological and pathological effects of PAF have been gradually identified (Cao et al., 2018). PAF receptor antagonists (PAFRAs) have different mechanisms of action compared to previous antiplatelet drugs and a variety of anti-inflammatory and neuroprotective effects (Vadas et al., 2008); they are currently widely used for blood circulation disorders and accumulating evidence has been shown them to be an effective treatment for ischemic cerebrovascular diseases (Wang et al., 2017). The postulated mechanism of action of PAFRAs is presented in Supplementary Material S1. PAFRAs are diverse and can be divided into two categories: natural and synthetic. Natural PAFRA components may be derived from plant, animal, or microbial fermentation sources, but medicinal plant compounds such as terpenoids, flavonoids, saponins, and other components are currently the main source of PAFRAs.

Ginkgo terpene lactones are terpenoid representatives of PAFRAs, extracted from *Ginkgo biloba* leaves. A number of different ginkgolides have been identified and labeled as A, B, C, K, J, L, M, N, P, and Q (Zeng et al., 2013). Among these, A, B, C, and K are the main active compounds of PAFRAs that are essential in treating cardiovascular and cerebrovascular diseases (Montrucchio et al., 2000). Injections of ginkgolides or the ginkgo diterpene lactone, meglumine, are the ginkgo terpene lactone-related preparations most widely used for AIS in clinical practice. Flavonoids have also received increasing interest for their potential effects on human health including their antioxidant, anti-inflammatory, lipid-modulating, and antiplatelet effects (Weseler and Bast, 2012). Hydroxysafflor yellow A (HYSA) and hesperidin are flavonoids with PAFRA activity. Hesperidin, the major flavanone glycoside in citrus fruits was found in a previous study to exhibit a protective effect against ischemic reperfusion cerebral injury in rats (Gaur et al., 2011). Studies have demonstrated that HYSA has neuroprotective effects in a cerebral ischemia model, in vivo and in vitro, through inhibition of platelet aggregation (Guo et al., 2019), and anti-inflammatory and antioxidant activity (Cheng et al., 2016), and by modulation of the autophagy pathway (Li et al., 2019). Ginsenosides are triterpenoid saponin representatives of PAFRAs found almost exclusively in ginseng that have been shown to dose-dependently inhibit the aggregation of washed platelets caused by thrombin and collagen (Jung et al., 1998). Although PAFRA-related preparations have shown good efficacy in experimental studies of AIS models, their main mode of activity and the corresponding add-on effects have not been systematically evaluated. Therefore, the aim of this systematic review was to summarize the evidence for treatment of AIS with PAFRAs, to facilitate a better understanding for patients as well as practitioners and provide guidance for rational clinical decision-making.

## 2 Methods

### 2.1 Search strategy

We searched several electronic databases including PubMed, Embase, Cochrane Library, Medline, China National Knowledge Infrastructure (CNKI), Wanfang Database, and China Science and Technology Journal Database (VIP). The systematic literature retrieval time ranged from the inception of each database to 11 March 2022. The details of the search strings used for each database are presented in the Supplementary Material S1. This systematic review was conducted based on the Preferred Reporting Items for Systematic reviews and Meta-analyses (PRISMA) (Radua, 2021) and focused on RCTs in patients with AIS undergoing PAFRA therapy within 7 days of stroke onset. Two independent reviewers (Xuebin Zhang and Tingting Li) screened the search results by reading titles and abstracts. If any disagreement arose between raters, the full-text was screened by two researchers (Dandan Zhang and Ping Jiang), and all disagreements were re-evaluated until consensus was achieved.

### 2.2 Selection criteria

Studies were included if they met the following criteria: **(1) Participants:** Participants included patients who met the World Health Organization (WHO) diagnostic criteria for AIS within 7 days after stroke onset, excluding cerebral hemorrhage demonstrated by brain computed tomography (CT) or magnetic resonance imaging (MRI). **(2) Intervention:** The experimental group comprised patients treated with PAFRA-related preparations alone or in combination with other therapies, which were referred to in the current guidelines (Powers et al., 2019) including antiplatelet therapy, control of vascular risk factors, and appropriate rehabilitation. **(3) Comparator:** The control group included patients treated with a placebo or other therapies. **(4) Outcomes:** The primary outcome of this study was modified Rankin Scale (mRS) score. Secondary outcomes included the National Institute of Health Stroke Scale (NIHSS) score and adverse events (AEs). **(5) Study design:** The study design included RCTs. The exclusion criteria were as follows: studies without sufficient available data; duplicate publications (only the first publication was included); and literature reviews, case reports, animal experiments, comments, editorials, congress abstracts, expert opinions, and articles that had not undergone peer review.

### 2.3 Data extraction and assessment of bias risk

The following data were extracted from each RCT independently by two reviewers (Tingting Li and Dandan Zhang): name of the first author, publication year, study period, demographic characteristics, sample size, intervention, treatment course, outcomes, and AEs. The methodological quality of the included studies was assessed with the Cochrane collaborations tool (Cumpston et al., 2019). Two trained researchers (Mingzhen Qin and Ping Jiang) independently evaluated the risk of bias of the included studies, namely, the random sequence generation, allocation concealment, blinding of participants and personnel, blinding of outcome assessment, incomplete outcome data, selective reporting, and other biases. Risk of bias was evaluated as low, high, and unclear.

### 2.4 Statistical analysis

The statistical package (RevMan 5.3) provided by the Cochrane collaboration was utilized to analyze the outcomes, including the mRS scores and the neurological functions as assessed by the NIHSS scores. Heterogeneity was evaluated using the chi-squared (Chi^2^) test and I-squared (I^2^) test. When the homogeneity was high (*p* ≥ 0.1, I^2^ <50%), the fixed-effects model was used. When there was heterogeneity in the results of the trials (*p* < 0.1, I^2^ ≥ 50%), the random-effects model was used for analysis. Continuous data were presented as the mean difference (MD), and dichotomous data were presented as odds ratio (OR) or the relative risk (RR) with 95% confidence intervals (95% CIs). Potential publication bias was examined by funnel plots with RevMan 5.3 software. To ascertain the causes of heterogeneity, and to determine whether or not the random effects model could be used for analysis, sensitivity or subgroup analyses were carried out. If there were too few studies to conduct a meta-analysis or if the clinical heterogeneity was too large, the descriptive analysis was used.

## 3 Results

### 3.1 Study selection process

**FIGURE 1:**
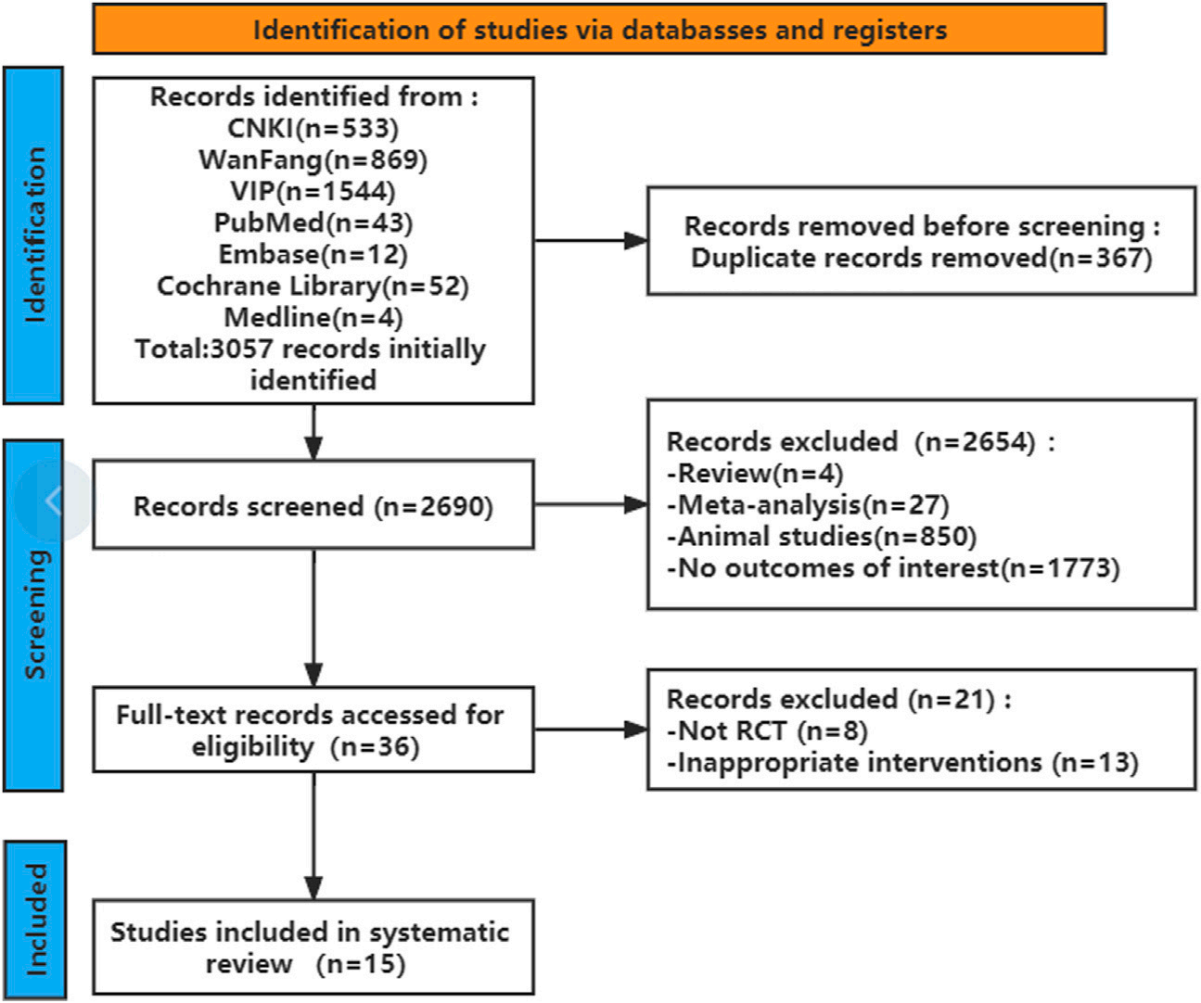
Study selection process for the systematic review.

A total of 2,690 articles were initially retrieved according to the search strategy. Of these, 367 articles were removed because of duplication. The remaining 2,323 articles were further filtered, and 2,654 articles were excluded according to the inclusion and exclusion criteria. After reading the full texts, 15 studies finally remained for systematic analysis (Figure 1). The characteristics of the included studies are provided in Table 1. There was a mix of comparisons of different PAFRAs with different comparators, but it was important not to combine outcomes that were too diverse.

**TABLE 1:** Overview of randomized controlled trials of platelet activating factor antagonists of natural origin for acute ischemic stroke.

Author	Sample size	Age	Symptom onset	Invention		Dose	Treatment course	Primary outcome	Secondary outcome	Adverse event	
	Exp/Con	Exp/Con		Exp	Con					Exp	Con
PAFRA + CM vs CM											
Su et al. (2018)	20/20	NR	<48 h	GEDM + CM	PLC + CM	5 ml, qd	90 days	mRS	BR,UR, NIHSS	NR	NR
Feng et al. (2020)	216/100	69.76 ± 11.70/68.49 ± 12.20	<48 h	GEDM + CM	CM	5 ml, qd	14 days	mRS	NIHSS, BI	NR	NR
Sun et al. (2019)	36/36	62.54 ± 3.98/60.18 ± 4.33	<72 h	GEDM + CM	CM	5 ml, qd	14 days	mRS	BR, NIHSS	Dizziness (1), gastrointestinal discomfort (1)	Gastrointestinal discomfort (2)
Zheng and Jiang (2018)	44/44	68.12 ± 12.83/69.45 ± 13.01	<72 h	GEDM + CM	CM	25 mg, qd	14 days	mRS	Fib, CPR, NIHSS	NR	NR
Huang et al. (2021)	54/54	51.23 ± 5.23/51.28 ± 5.32	<48 h	GEDM + CM	CM	25 mg, qd	14 days	mRS	NSE, hs-CRP	Rash (1), itching (3)	Skin rash (1), skin itching (2)
Dong 2021	463/473	64.31 ± 10.68/64.12 ± 10.40	<72 h	GDI + CM	PLC + CM	10 ml, qd	14 days	Mortality, stroke recurrence	mRS, NIHSS, PAF, ADP, TAX2	Major bleeding events (5)	Major bleeding events (3)
Ren 2020	53/53	59.71 ± 6.73/59.63 ± 6.72	<48 h	GBDP + CM	CM	8 pills, tid	14 days	mRS	BR, NIHSS, BI	Vascular pain (1), prolonged clotting time (1), stomach discomfort (1)	Vascular pain (1), prolonged clotting time (1), abnormal liver function (1)
PAFRA + CM + NPA vs CM + NPA											
Liu (2018)	20/20	58.12 ± 13.28/56.26 ± 14.36	24–72 h	GDI + NBP + CM	NBP + CM	10 ml, qd	14 days	mRS	ADL, NIHSS	NR	NR
Bao and Peng (2019)	65/63	64.50 ± 5.50/66.50 ± 5.00	<12 h	GEDM + EDV + CM	EDV + CM	20 mg, qd	14 days	mRS	NIHSS	NR	NR
Wang 2017	40/40	75.5 ± 5.25/76.6 ± 5.25	<72 h	GEDM + EDV + CM	EDV + CM	25 mg, qd	14 days	mRS	NIHSS	NR	NR
PAFRA + CM + IVT vs CM + IVT											
Zhou (2021)	40/40	61.58 ± 3.19/61.65 ± 3.27	<6 h	GDI + rt-PA + CM	rt-PA + CM	6 ml, qd	14 days	mRS	BFR, NIHSS	NR	NR
Zhang 2021	513/600	68 ± 12/68 ± 12	<4.5 h	GDI + rt-PA + CM	rt-PA + CM	10 ml, qd	14 days	mRS	NIHSS	NR	NR
Qin 2019	171/170	62.4 ± 8.1/62.5 ± 7.9	<3 h	HES + rt-PA + CM	rtPA + CM	4 mg/kg	7 days	mRS	TGF-β1,MMP-2,MMP-9, TCD, NIHSS 7	Fatal symptomatic hemorrhage (2), coagulopathy (8)	Symptomatic hemorrhage fatal (6), nonfatal (3), coagulopathy (10)
Liu et al. (2020)	90/90	58.62 ± 5.23/60.45 ± 5.09	<4.5 h	GSRI + rt-PA + CM	rt-PA + CM	10 mg, qd	14 days	mRS	FMA, PAdT, PAgT, CD62p, CD63, NIHSS, BI	Deterioration (3)	Deterioration (20)
PAFRA + CM vs. other herbal extracts + CM											
Hu 2020	208/71	NR	<7 days	HSYA + CM	DZXXI + CM	25, 50, and 70 mg/d	14 days	mRS	NIHSS	Gastrointestinal disorders: low-dose group (8), high-dose (6)	Hepatobiliary disorders (6)

Note: GEDM, ginkgo endoterpene diester meglumine; GDI, ginkgolide injection; GBDP, ginkgo bilobate dropping pill; HES, hesperidin; GSRI, ginsenoside Rd injection; HSYA, hydroxysafflor yellow A; CM, conventional treatment; NBP, n-butylphthalide; EDV, edaravone; rt-PA, recombinant tissue plasminogen activator; DZXXI, Dengzhan Xixin injection; PLC, placebo; BR, blood routine; UR, urine routine; NIHSS, National Institute of Health stroke scale; mRS, modified Rankin scale; NR, not reported; BFR, blood flow rate; ADL, activities of daily living; NSE, neuron-specific enolase; hs-CRP, hypersensitive C-reactive protein; Fib, fibrinogen; FMA, Fugl-Meyer scale; PAdT, platelet adhesion test; PAgT, platelet aggregation test; CD62p, CD63, granule membrane glycoprotein; TGF-β1, transforming growth factor; MMP-2, MMP-9, matrix metalloproteinases; TCD, transcranial Doppler; ADP, adenosine-5′-diphosphate; PAF, platelet aggregation factor; TXA2, thromboxane A2.

### 3.2 Risk of bias within studies

**FIGURE 2:**
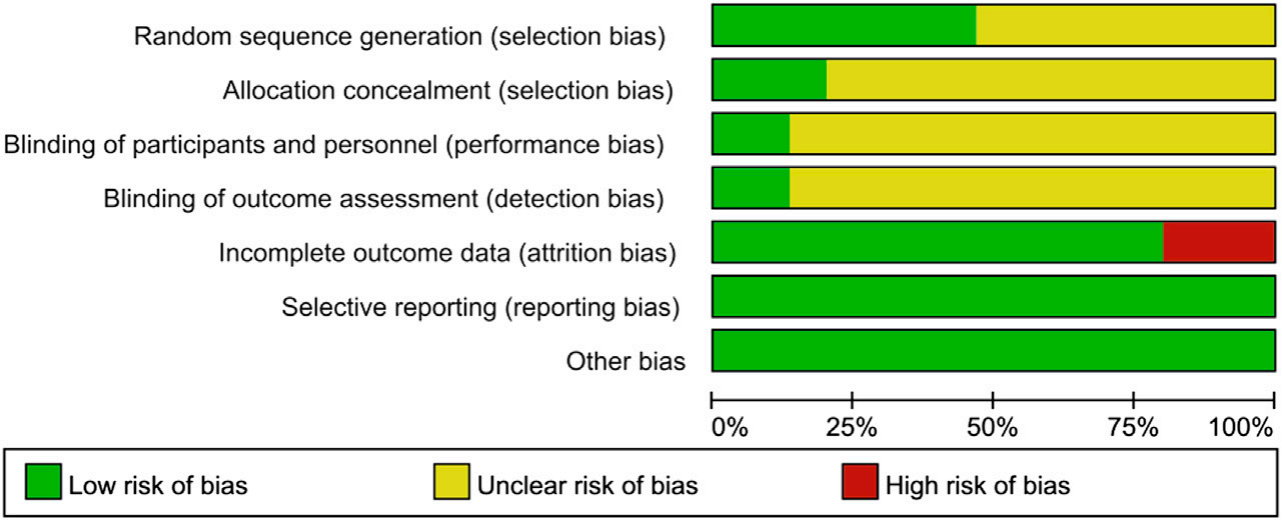
Risk of bias graph. The judgments of the reviewing authors about each domain of bias are presented as percentages of all included studies. The quality of the selected studies was assessed according to the Cochrane criteria.

**FIGURE 3:**
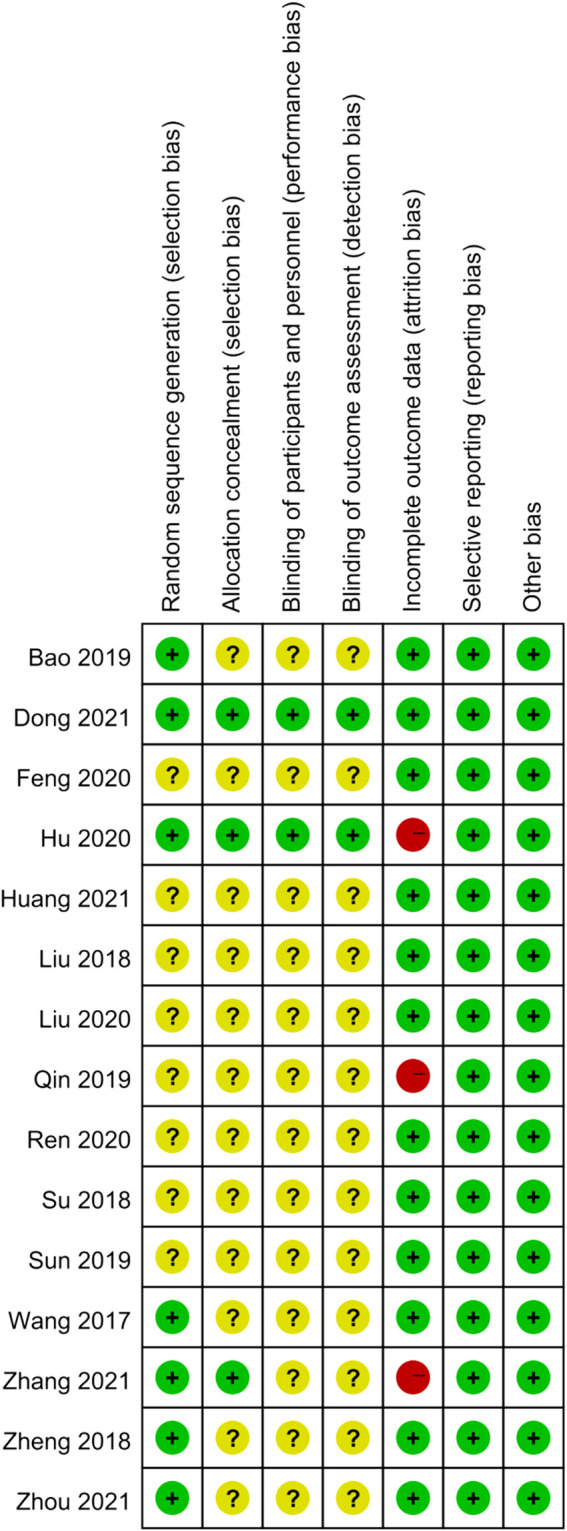
Risk of bias summary. The judgments of the reviewing authors about each domain of bias for each included study were summarized.

The results of the risk of bias assessment were as follows. In terms of random sequence generation, only seven RCTs described the specific randomization method, so they were evaluated as low risk. As for allocation concealment, only three of the trials described a random allocation plan. Regarding performance bias, only two double-blind studies were evaluated as low risk, and the other studies did not mention blinding. In terms of attrition bias, only two studies mentioned the blinding of the evaluators. Three studies with incomplete outcome data were rated as high risk. None of the articles described the other risk biases. The results of the risk bias assessment are presented in Figures 2,3.

### 3.3 Basic characteristics of included studies

Fifteen RCTs comprising a total of 3,907 participants were included in this study (2033 in the experimental group and 1874 in the control group). There were no significant differences in sex, age, or course of disease between the study groups, with comparable baselines. Most RCTs focused on GEDM (7 RCTs, 46.6%) (Su et al., 2018; Feng et al., 2020; Sun et al., 2019; Zheng and Jiang, 2018; Huang et al., 2021; Bao and Peng, 2019; Wang et al., 2017), followed by GDI (4 RCTs, 26.6%) (Dong et al., 2021; Zhang et al., 2021; Zhou, 2021; Liu, 2018), GBDP (1 RCT, 6.7%) (Sun et al., 2018), HES (1 RCT, 6.7%) (Qin et al., 2020), GSRI (1RCT, 6.7%) (Liu et al., 2020), and HSYA (1 RCT, 6.7%) (Hu et al., 2020). The course of treatment ranged from 7 days to 90 days. The detailed characteristics of the fifteen trials are summarized in Table 1.

### 3.4 Studies combining PAFRA with conventional treatment

#### 3.4.1 Ginkgo endoterpene diester meglumine (GEDM)

GEDM is composed of ginkgolides A, B, and K, which are the main active compounds of PAFRAs. Five trials investigated the use of GEDM in patients with AIS with CM, and one trial (Su et al., 2018) used a placebo-controlled design to rule out the placebo effect as a partial explanation for differences between groups. Three trials measured long-term outcomes on D90 using the mRS score. The mRS scores of patients in the GEDM combined with CM group on D90 were lower than those of patients in the group that received CM alone, and the difference was statistically significant (*p* < 0.05) (Su et al., 2018; Feng et al., 2020; Sun et al., 2019). However, one trial (Zheng and Jiang, 2018) found no difference in the mRS scores on D90 between the two groups (*p* > 0.05). Moreover, one trial (Huang et al., 2021) reported positive results with lower mRS scores on D60 in the GEDM-treated group than in the CM group. In total, five articles reported that the mRS scores showed no statistical heterogeneity (*p* = 0.64, I^2^ = 0%); thus, the fixed-effects model was employed for analysis. The results showed that the mRS scores of patients treated with GEDM combined with CM was lower than those of patients treated with CM alone [overall MD = −0.42, 95% CI (−0.47, −0.37), five trials, *p* < 0.00001]. The forest plot and publication bias figure of GEDM combined with CM for AIS on mRS are presented in Supplementary Material S1. With regard to neurological function deficits, four articles reported the NIHSS scores with no statistical heterogeneity (*p* = 0.52, I^2^ = 0%); therefore, the fixed-effects model was utilized. The results showed that GEDM combined with CM was superior to CM alone in improving neurological function [overall MD = −1.02, 95% CI (−1.51, −0.52), four trials, *p* < 0.0001]. AEs were reported in one study (Sun et al., 2019)and included one case of dizziness, one case of gastrointestinal discomfort in the GEDM group, and two cases of gastrointestinal discomfort in control group; no statistically significant differences were seen between the groups (*p* > 0.05) (Tables 2,3).

**TABLE 2:** Effect of platelet-activating factor antagonists on mRS in acute ischemic stroke.

Comparison	Outcome		Experimental (mean ± SD)/(n/N)	Control (mean ± SD)/(n/N)	Relative benefit (95% CI)	*p* Value	References
PAFRA + CM vs CM							
GEDM + CM vs CM	90D mRS		0.95 ± 0.87	1.32 ± 1.04	−0.37 [−0.77, 0.03]	*p* = 0.07	Zheng and Jiang (2018)
	90D mRS		1.53 ± 0.97	2.18 ± 1.13	−0.65 [−1.14, −0.16]	*p* = 0.009	Sun et al. (2019)
	90D mRS		0.6 ± 0.49	1.35 ± 1.15	−0.75 [−1.30, −0.20]	*p* = 0.007	Su et al. (2018)
	60D mRS		0.61 ± 0.13	1.02 ± 0.14	−0.41 [−0.46, −0.36]	*p* < 0.00001	Huang et al. (2021)
	90D mRS		2.41 ± 1.16	2.86 ± 0.91	−0.45 [−0.69, −0.21]	*p* = 0.0002	Feng et al. (2020)
		Overall			−0.42 [−0.47, −0.37]	*p* < 0.00001	
GDI + CM vs PLC + CM	Proportion of mRS (28D)≤2		362/463	362/473	1.49 [1.01, 2.20]	*p* = 0.042	Dong 2021
GBDP + CM vs CM	14D mRS		1.63 ± 0.17	2.38 ± 0.24	−0.75 [−0.83, −0.67]	*p* < 0.00001	Ren 2020
PAFRA + CM + NPA vs CM + NPA							
GEDM + EDV + CM vs. EDV + CM	14D mRS		2.0 ± 0.5	2.4 ± 0.5	−0.40 [−0.57, −0.23]	*p* < 0.00001	Bao and Peng (2019)
	14D mRS		2.05 ± 0.55	2.45 ± 0.55	−0.40 [−0.64, −0.16]	*p* = 0.001	Wang 2017
		Overall			−0.40 [−0.54, −0.26]	*p* < 0.00001	
GDI + NBP + CM vs. NBP + CM	14D mRS		2 ± 0.65	2.6 ± 0.68	−0.60 [−1.01, −0.19]	*p* = 0.004	Liu (2018)
PAFRA + CM + IVT vs CM + IVT							
GDI + rt-PA + CM vs. rt-PA + CM	14D mRS		2.68 ± 0.67	3.78 ± 0.92	−1.10 [−1.45, −0.75]	*p* < 0.00001	Zhou (2021)
	Proportion of mRS (90D)≤2		311/404	285/404	1.09 [1.00, 1.19]	*p* = 0.04	Zhang 2021
HES + rt-PA + CM vs. PLC + rt-PA + CM	Proportion of mRS (2 M)≤1		80/171	74/170	1.07 [0.85, 1.36]	*p* = 0.55	Qin 2019
GSRI + rt-PA + CM vs. rt-PA + CM	Proportion of mRS (14D)≤1		62/90	38/90	1.63 [1.23, 2.16]	*p* = 0.0006	Liu et al. (2020)
PAFRA + CM vs. other herbal extracts + CM							
HSYA + CM vs. DZXXI + CM	Proportion of mRS (90D)≤1	Low-dosage	22/66	26/68	0.87 [0.55, 1.38]	*p* = 0.56	Hu 2020
	Proportion of mRS (90D)≤1	Medium-dosage	38/67	26/68	1.48 [1.03, 2.14]	*p* = 0.04	Hu 2020
	Proportion of mRS (90D)≤1	High-dosage	38/65	26/68	1.53 [1.06, 2.20]	*p* = 0.02	Hu 2020

Note: GEDM, ginkgo endoterpene diester meglumine; GDI, ginkgolide injection; GBDP, ginkgo bilobate dropping pill; HES, hesperidin; GSRI, ginsenoside Rd injection; HSYA, hydroxysafflor yellow A; CM, conventional treatment; NBP, n-butylphthalide; EDV, edaravone; rt-PA, recombinant tissue plasminogen activator; DZXXI, Dengzhan Xixin injection; PLC, placebo.

**TABLE 3:** Effect of platelet-activating factor antagonists on NIHSS in acute ischemic stroke.

Comparison	Outcome		Experimental (mean ± SD)/(n/N)	Control (mean ± SD)/(n/N)	Relative benefit (95% CI)	*p* Value	References
PAFRA + CM vs CM							
GEDM + CM vs CM	14D NIHSS		2.10 ± 2.09	2.68 ± 2.53	−0.58 [−1.55, 0.39]	*p* = 0.24	Zheng and Jiang (2018)
	14D NIHSS		7.90 ± 3.16	8.62 ± 3.24	−0.72 [−2.20, 0.76]	*p* = 0.34	Sun et al. (2019)
	14D NIHSS		1.7 ± 1.6	3.3 ± 1.6	−1.60 [−2.59, −0.61]	*p* = 0.002	Su et al. (2018)
	NA		NA	NA	NA	NA	Huang et al. (2021)
	14D NIHSS		5.62 ± 2.77	6.64 ± 3.66	−1.02 [−1.83, −0.21]	*p* = 0.01	Feng et al. (2020)
		Overall			−1.02 [−1.51, −0.52]	*p* < 0.0001	
GDI + CM vs. PLA + CM	28D NIHSS improvement		3.73 ± 2.24	3.36 ± 2.28	0.37 [0.07, 0.68]	*p* = 0.016	Dong 2021
GBDP + CM vs CM	14D NIHSS		4.52 ± 0.92	7.59 ± 1.39	−3.07 [−3.52, −2.62]	*p* < 0.00001	Ren 2020
PAFRA + CM + NPA vs CM + NPA							
GEDM + EDV + CM vs. EDV + CM	14D NIHSS		5.65 ± 1.05	11.52 ± 2.50	−5.87 [−6.54, −5.20]	*p* < 0.00001	Bao and Peng (2019)
	14D NIHSS		5.66 ± 1.15	7.66 ± 1.15	−2.00 [−2.50, −1.50]	*p* < 0.00001	Wang 2017
		Overall			−3.93 [−7.72, −0.14]	*p* = 0.04	
GDI + NBP + CM vs. NBP + CM	14D NIHSS		3.42 ± 1.95	4.79 ± 2.04	−1.37 [−2.61, −0.13]	*p* = 0.03	Liu (2018)
PAFRA + CM + IVT vs CM + IVT							
GDI + rt-PA + CM vs. rt-PA + CM	14D NIHSS		6.12 ± 0.85	9.35 ± 1.17	−3.23 [−3.68, −2.78]	*p* < 0.00001	Zhou (2021)
	ENI (7D)		321/423	282/423	1.14 [1.04, 1.24]	*p* = 0.003	Zhang 2021
HES + rt-PA + CM vs PLC + rt-PA + CM	Proportion of NIHSS(2 M)≤1		75/171	73/170	1.02 [0.80, 1.30]	*p* = 0.86	Qin 2019
GSRI + rt-PA + CM vs. rt-PA + CM	14D NIHSS		10.03 ± 1.29	18.90 ± 2.42	−8.87 [−9.44, −8.30]	*p* < 0.00001	Liu et al. (2020)
PAFRA + CM vs. other herbal extracts + CM							
HSYA + CM vs. DZXXI + CM	NA		NA	NA	NA	NA	Hu 2020

Note: GEDM, ginkgo endoterpene diester meglumine; GDI, ginkgolide injection; GBDP, ginkgo bilobate dropping pill; HES, hesperidin; GSRI, ginsenoside Rd injection; HSYA, hydroxysafflor yellow A; CM, conventional treatment; NBP, n-butylphthalide; EDV, edaravone; rt-PA, recombinant tissue plasminogen activator; DZXXI, Dengzhan Xixin injection; PLC, placebo; ENI, neurological improvement defined as (NIHSS_baseline_-NIHSS_7D_)/NIHSS_baseline_*100% ≥ 18%; NIHSS improvement: NIHSS_baseline_-NIHSS_28D_.

#### 3.4.2 Ginkgolide injection (GDI)

GDI contains bilobalide and ginkgolides A, B, and C, which are commonly used in the clinical treatment of stroke in China to promote blood circulation and eliminate blood stasis. In one multi-center, randomized, double-blinded, placebo-controlled trial (Dong et al., 2021) patients diagnosed with AIS with intracranial artery stenosis (ICAS) were assigned to either GDI or placebo treatment. The trial reported a favorable outcome with a greater proportion of mRS scores ≤2 on D28 and NIHSS score improvement (NIHSS_baseline_-NIHSS_28D_) in the GDI-treated group compared with the placebo group [RR_mRS_ = 1.49, 95% CI (1.01, 2.20), *p* = 0.042; MD_NIHSS improvement_ = 0.37, 95% CI (0.07, 0.68), *p* = 0.016]. Serious AEs (SAEs) occurred in five patients in the GDI and three in placebo groups, respectively [RR = 1.703, 95% CI (0.40, 7.08), *p* = 0.502] (Tables 2,3).

#### 3.4.3 Ginkgo bilobate dropping pill (GBDP)

GBDP is a unique *G. biloba* leaf extract produced in China with antioxidant and neuroprotective effects in various conditions (Sun et al., 2018). Quantitative analysis showed that EGb761, a standardized *G. biloba* leaf extract, had higher levels of organic acids than GBDP, while GBDP had higher levels of flavonoids (Zhang et al., 2022). One trial investigated the use of GBDP with CM, tested in 106 patients with AIS (Ren et al., 2020). The researchers reported positive results with lower mRS scores and NIHSS scores on D14 in the GBDP-treated group than in the CM group [MD_mRS_ = −0.75, 95% CI (−0.83, −0.67), *p* < 0.00001; MD_NIHSS_ = −3.07, 95% CI (−3.52, −2.62), *p* < 0.00001]. With regard to the AEs, the GBDP-treated group reported one case of vascular pain, one case of prolonged coagulation time, and one case of gastric discomfort, while the control group reported one case of vascular pain, one case of prolonged coagulation time, and two cases of abnormal liver function; there were no significant differences in the incidence of AEs (5.66 vs 7.55%, *p* > 0.05) (Tables 2,3).

### 3.5 Studies combining PAFRA with neuroprotective agents

#### 3.5.1 Ginkgo endoterpene diester meglumine

In two trials, Bao and Peng (2019) and Wang et al. (2017) investigated the application of GEDM in patients with AIS receiving the neuroprotective agent (NPA), edaravone (EDV). There was no statistical heterogeneity (*p* = 1.00, I^2^ = 0%), and therefore, the fixed-effects model was employed for analysis. The results were positive with lower mRS scores and NIHSS scores on D14 in the GEDM- plus EDV-treated group than in the EDV group [MD_mRS_ = −0.40, 95% CI (−0.54, −0.26), *p* < 0.00001]. Considering the high heterogeneity in the meta-analysis of the NIHSS scores (*p* < 0.00001, I^2^ = 99%), sensitivity analysis should be performed to determine the sources and confounding factors of heterogeneity and to determine the robustness of the review results. However, we were unable to conduct the planned sensitivity analysis due to the small number of included studies (two); therefore, the random-effects model was employed for analysis. The results were positive with lower NIHSS scores on D14 in the GEDM- plus EDV-treated group than in the EDV group [MD_NIHSS_ = −3.93, 95%CI (−7.72, −0.14), *p* = 0.04]. No AEs were reported (Tables 2,3). The forest plots of GEDM plus EDV for AIS on mRS or NIHSS are shown in Supplementary Material S1.

#### 3.5.2 Ginkgolide injection

One trial (Liu, 2018) investigated the application of GDI in patients with AIS receiving the NPA of n-butylphthalide (NBP). Treatment with GDI combined with NBP showed a significantly better effect on D14 than treatment with NBP alone in terms of mRS and NIHSS scores [MD_mRS_ = −0.60, 95% CI (−1.01, −0.19), *p* = 0.004; MD_NIHSS_ = −1.37, 95%CI (−2.61, −0.13), *p* = 0.03](Tables 2,3).

### 3.6 Studies combining PAFRA with thrombolytic therapy

#### 3.6.1 Ginkgolide injection

Two trials (Zhang et al., 2021; Zhou, 2021) were done to investigate the use of GDI in patients with stroke receiving intravenous (IV) recombinant tissue plasminogen activator (rt-PA). The GIANT study (Zhang et al., 2021), a cluster-randomized trial, included patients with AIS from 24 centers, assigned to an intervention of IV GDI with rt-PA or a control group within the first 24 h after IV rt-PA therapy (IVT). Patients in the GDI group were more likely to have good outcomes, defined as the proportion of patients with an mRS scores ≤2 at 90 days [RR = 1.09, 95% CI (1.00, 1.19), *p* = 0.04], than patients in the control group. With regard to the neurological improvement (ENI), which was defined as (NIHSS_baseline_-NIHSS_7D_)/NIHSS_baseline_*100% ≥ 18% at 7 days, the intervention with GDI resulted in a significantly better ENI [RR = 1.14, 95% CI (1.04, 1.24), *p* = 0.003] than the control group. No significant differences in AEs of hemorrhage transformation were seen between groups [RR = 0.885; 95% CI (0.450, 1.741), *p* = 0.724]. The other trial (Zhou, 2021) reported positive results with lower mRS and NIHSS scores on D14 in the GDI plus IVT-treated group than the IVT group [MD_mRS_ = −1.10, 95% CI (−1.45, −0.75), *p* < 0.00001; MD_NIHSS_ = −3.23, 95% CI (−3.68, −2.78), *p* < 0.00001] (Tables 2,3).

#### 3.6.2 Hesperidin

Hesperidin is the major flavanone glycoside in citrus fruits, and a flavonoid representative of PAFRAs. In one trial (Qin et al., 2020), patients with AIS were randomly assigned to two groups to receive either rt-PA plus hesperidin or rt-PA plus placebo (PLC). Follow-up hesperidin treatment for seven consecutive days was found to improve the long-term favorable recovery of neurological function, as indicated by the proportion of scores in the range of 0–1 for mRS and NIHSS after 2 months. However, the difference was not significant [RR_mRS_ = 1.07, 95% CI (0.85, 1.36), *p* = 0.55; RR_NIHSS_ = 1.02, 95% CI (0.80, 1.30), *p* = 0.86]. Co-treatment with HES yielded significant improvement by decreasing the incidence of symptomatic intracerebral hemorrhage (SIH) following rt-PA [hesperidin group vs control group:9/170 vs. 2/171] (Tables 2,3).

#### 3.6.3 Ginsenoside Rd injection

Ginsenosides are triterpenoid saponins representative of PAFRAs that have been shown to improve the microcirculation by inhibiting the aggregation of washed platelets. Liu et al. (2020) investigated 180 patients with AIS, who received randomized treatment with GSRI plus IVT or IVT for 2 weeks. This study reported positive results with lower NIHSS scores on D14 in the GSRI plus IVT-treated group than in the IVT group [MD_NIHSS_ = −8.87, 95% CI (−9.44, −8.30), *p* < 0.00001]. Patients in the GSRI group were more likely to have good outcomes, which were defined as a higher proportion of patients with mRS scores ≤1 at 14 days [RR = 1.63, 95% CI (1.23, 2.16), *p* = 0.0006], than patients in the control group. No AEs were reported (Tables 2,3).

### 3.7 Hydroxysafflor yellow A injection

HSYA was isolated from the dried flowers of *Carthamus tinctorius* L. and possesses some important medicinal activities such as blood circulation activation and elimination of blood stasis (Tian et al., 2010). In a trial of HSYA (Hu et al., 2020), 288 patients with AIS were randomly assigned to low-, medium-, and high-dose HSYA groups (25, 50, and 70 mg/d HSYA, respectively) and a control group DZXXI for 14 consecutive days. Significantly more patients in the medium- and high-dose HSYA groups than in the control group had an mRS score less than one on D90 [RR_medium_ = 1.48, 95% CI (1.03, 2.14), *p* = 0.04; RR_high_ = 1.53, 95% CI (1.06, 2.20), *p* = 0.02]. No significant difference was seen among groups in AEs (*p* > 0.05) (Table 2).

## 4 Discussion

### 4.1 Scientific implications and innovations

Due to the severity of acute stroke, the limited therapeutic options, and the unfavorable outcomes, there is a critical need for new therapies to improve function in patients with AIS. Patients with AIS have been shown to have overactive PAF, a powerful inducer of in vivo platelet activation and thrombosis (Chignard et al., 1979). In addition to platelet activation, PAF has been found to directly damage neurons, through brain vasoconstriction and hypoperfusion, which may be induced by particular PAF binding sites in the brain (Domingo et al., 1988; Toscano et al., 2016; Zhao et al., 2021). Although the beneficial effects of aspirin in the treatment and prevention of AIS are well known, the antiplatelet effects are limited because the inhibition of cyclooxygenase by aspirin does not suppress platelet responsiveness to all in vivo thrombogenic stimuli (Joseph et al., 1989). PAF has been reported to activate platelets independently of the cyclooxygenase pathway (Shah et al., 2001), which means that the platelet aggregation induced by PAF in AIS patients could not be suppressed by aspirin. Therefore, in order to prevent thrombosis effectively, the suppression of PAF-induced platelet activation may involve more activities in addition to those provided by collagen and thrombin.

The first 4.5 h after AIS are considered the “golden period” for receiving thrombolytic therapy. IVT not only disrupted the blood–brain barrier (BBB) but also resulted in cytotoxic brain edema (Yepes et al., 2009). Over the past few years, PAFRAs of natural origin have been studied for a wide variety of neurological and neuropsychiatric conditions. Notably, the just-mentioned study indicated that the effective derivative isolated from an herb like *Ginkgo biloba* could exert a mitigating effect on disorders in neurotransmitter metabolism, energy, and amino acids caused by rt-PA (Chen et al., 2018). Therefore, whether PAFRA-related preparations could be used as complementary therapies to decrease the incidence of AEs caused by IVT and improve their clinical efficacy is also crucial and promising. Additionally, *Ginkgo biloba* has been demonstrated to have both in vitro and in vivo selective PAF-antagonistic effects on human platelets (Chung et al., 1987; Koch, 2005). A recent expert consensus (Immunology Society of Chinese Stroke Society, 2020) recommended PAFRA as a therapeutic measure for AIS, which has contributed to its widespread use among patients with AIS. Because of that, further investigation of the safety and synergistic effects on platelet inhibition of the combination of different kinds of derivatives of medicinal plants with PAF-antagonistic properties and other therapies has important clinical implications. Our study has taken the lead in conducting a systematic review to investigate the effects and safety of six PAFRA-related preparations in the treatment of AIS.

### 4.2 Summary of main results

This systematic review covered twelve studies that used *G. biloba* extracts to treat AIS: seven studies of GEDM, one study of GBDP, four studies of GDI, and one study each of HES, HSYA, and GSRI. In terms of clinical efficacy, the combination therapy of PAFRA-related preparations including GEDM, GDI or GBDP plus CM treatment was superior to CM alone in improving the functional outcomes of mRS and NIHSS scores. Furthermore, a beneficial effect was noted on the clinical efficacy of AIS with the combination therapy of GEDM + EDV + CM or GDI + NBP + CM in comparison with NPA + CM. Thus, combination therapy of PAFRA-related preparations with NPA might be an attractive option for the treatment of AIS. In terms of safety, no significant differences of GEDM, GDI, or GBDP with regard to AEs were found. Moreover, the study of HSYA (Hu et al., 2020), a head-to-head comparison, found greater clinical efficacy with medium and high doses of HSYA + CM (50 and 70 mg/d, respectively) compared with DZXXI + CM. AEs did not differ significantly between the two groups.

In this systematic review, there were five studies about efficacy and safety of PAFRA-related preparations combined with rt-PA in the treatment of AIS: two studies of GDI, one of GSRI, and one of HES. The combination therapy of GDI + rt-PA was superior to rt-PA alone in improving the clinical efficacy, and one open-label, prospective, multicenter cluster RCT (Zhang et al., 2021) also found that the administration of GDI after IVT did not involve a higher risk of hemorrhage transformation or symptomatic intracerebral hemorrhages. Simultaneously, the combination of GSRI + rt-PA was found to be superior to rt-PA alone in the treatment of AIS. However, after IVT for 7 consecutive days, HES treatment did not lead to significant improvements in the long-term favorable recovery of neurological function, as indicated by the proportion of scores of 0–1 on the mRS and NIHSS after 2 months. Interestingly, the study of Qin et al. (2020) suggested the potential clinical application of HES with rt-PA therapies to reduce SIH. It is worth noting that GSRI and HES had only one study each; therefore, we suggest being cautious in understanding and interpreting the results, until further confirmation is done to elucidate these findings.

Recurrent strokes are more likely to be disabling or even fatal in comparison with first-time strokes (Coull and Rothwell, 2004), but little attention has been paid to recurrent strokes. Determining the risk of recurrent strokes from the perspective of etiologic subgroups has particular clinical implications. One study included in this systematic review (Dong et al., 2021) targeted GDI intervention to a specific group of AIS patients with intracranial artery stenosis (ICAS) and recorded the rate of stroke recurrence and mortality at day 28 after randomization. The results showed that GDI was associated with fewer recurrent strokes in patients given ICAS [0/463 vs 6/473,RR = 1.013, 95% CI (1.003, 1.023), *p* = 0.031]. No patients died in either of the groups. However, this study did not analyze the influence on the recurrence caused by confounders, such as the residual flow, leptomeningeal collateral flow, or hemispheric blood flow. As such, this systematic review suggests that more well-designed studies are required to demonstrate the long-term outcomes of recurrence and effects in the field of stroke treatment.

### 4.3 Strengths and limitations

Despite the fact that several meta-analyses have been published (Chong et al., 2020; Zhao S. et al., 2021) on the efficacy and safety of ginkgo leaf extract preparations, the results should be interpreted with caution as the quality of the evidence is weak, which limits the applicability. Additionally, the studies included in those meta-analyses had a very short follow-up period, which was insufficient to assess changes to neurological functional outcomes like mRS. In general, the NIHSS score is used as the primary outcome for most systematic reviews. However, unlike most studies, this systematic review applied the internationally recognized functional outcome, namely, mRS, as one of the efficacy outcomes to assess the therapeutic value of PAFRA-related preparations. In terms of limitations, the majority of the fifteen studies had relatively low methodological quality on average, which provided only limited descriptions of the generation of the allocation sequence and the allocation concealment, and only two were double blinded. Despite the improved scientific rigor of our study design compared with earlier studies, the evidence from the limited number of high-quality studies is insufficient to identify all the gaps, differences, and trends of the various kinds of PAFRA preparations. Thus, caution should be used in interpreting these results. Also, the ginkgo leaf extract used here was also produced and tested in China, which may result in unavoidable regional bias and limited generalizability.

## 5 Conclusion

This systematic review summarized the evidence regarding the efficacy and safety of PAFRA interventions in terms of functional outcomes in patients with acute ischemic stroke and highlighted the need for adequately powered, well-designed, clinical trials to increase the quality of the available evidence. The summary of findings also suggested that researchers should measure and report core functional outcomes in future PAFRA clinical studies.

## Data Availability

The original contribution presented in the study are included in the article/Supplementary Material; further inquiries can be directed to the corresponding authors.
