# Supplementary material for: Platelet-activating factor receptor antagonists of natural origin for acute ischemic stroke: a systematic review of current evidence
Source: Front Pharmacol. 2022 Aug 31;13:933140. doi: 10.3389/fphar.2022.933140 (PMC9471864; doi:10.3389/fphar.2022.933140)

**Supplementary Files**

**Title:** Platelet-Activating Factor Receptor Antagonists of Natural Origin for Acute Ischemic Stroke: A Systematic Review of Current Evidence

### Section I

### Preferred Reporting Items for Systematic Reviews and Meta-Analyses (PRISMA) 2020 checklist

- **Section II**

Searching strategies

- **Section III**

Appendix Figure1.Chemical structure of Platelet-Activating Factor Receptor Antagonists of Natural Origin

- **Section IV**

Appendix Table1. Patented formulations,botanical or chemical

- **Section V**

Appendix Figure 2. Forest plot of Ginkgo Endoterpene Diester Meglumine combined with conventional medicine on mRS.

Appendix Figure 3. Forest plot of Ginkgo Endoterpene Diester Meglumine combined with conventional medicine on NIHSS.

Appendix Figure 4. Forest plot of Ginkgo Endoterpene Diester Meglumine combined with edaravone on mRS.

Appendix Figure 5. Forest plot of Ginkgo Endoterpene Diester Meglumine combined with edaravone on NIHSS.

Appendix Figure 6. Forest plot of Ginkgo Endoterpene Diester Meglumine combined with other therapies for AIS on mRS (different types of invention).

Appendix Figure 7. Forest plot of Ginkgo Endoterpene Diester Meglumine combined with other therapies for AIS on NIHSS (different types of invention).

Appendix Figure 8. Funnel plot of Ginkgo Endoterpene Diester Meglumine combined with other therapies for AIS on mRS.

- **Section VI**

Editorial Certificate

- **Section VII**

Appendix Figure 9. Platelet-Activating Factor Receptor Antagonists mechanism of action.


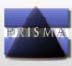
 **Section I. PRISMA 2020 Checklist**

| **Section and Topic** | **Item #** | **Checklist item** | **Location where item is reported** |
| --- | --- | --- | --- |
| **TITLE** | | |  |
| Title | 1 | Identify the report as a systematic review. | Page 1, line 1 |
| **ABSTRACT** | | |  |
| Abstract | 2 | See the PRISMA 2020 for Abstracts checklist. | Page 3, line 26 |
| **INTRODUCTION** | | |  |
| Rationale | 3 | Describe the rationale for the review in the context of existing knowledge. | Page 5, line 70 |
| Objectives | 4 | Provide an explicit statement of the objective(s) or question(s) the review addresses. | Page 6, line 133 |
| **METHODS** | | |  |
| Eligibility criteria | 5 | Specify the inclusion and exclusion criteria for the review and how studies were grouped for the syntheses. | Page 7, line 151 |
| Information sources | 6 | Specify all databases, registers, websites, organisations, reference lists and other sources searched or consulted to identify studies. Specify the date when each source was last searched or consulted. | Page 6, line 138 |
| Search strategy | 7 | Present the full search strategies for all databases, registers and websites, including any filters and limits used. | Supplementary Files  Section II |
| Selection process | 8 | Specify the methods used to decide whether a study met the inclusion criteria of the review, including how many reviewers screened each record and each report retrieved, whether they worked independently, and if applicable, details of automation tools used in the process. | Page 7, line 169 |
| Data collection process | 9 | Specify the methods used to collect data from reports, including how many reviewers collected data from each report, whether they worked independently, any processes for obtaining or confirming data from study investigators, and if applicable, details of automation tools used in the process. | Page 7, line 173 |
| Data items | 10a | List and define all outcomes for which data were sought. Specify whether all results that were compatible with each outcome domain in each study were sought (e.g. for all measures, time points, analyses), and if not, the methods used to decide which results to collect. | Page 8, line 182 |
|  | 10b | List and define all other variables for which data were sought (e.g. participant and intervention characteristics, funding sources). Describe any assumptions made about any missing or unclear information. | Page 8, line 188 |
| Study risk of bias assessment | 11 | Specify the methods used to assess risk of bias in the included studies, including details of the tool(s) used, how many reviewers assessed each study and whether they worked independently, and if applicable, details of automation tools used in the process. | Page 7, line 169 |
| Effect measures | 12 | Specify for each outcome the effect measure(s) (e.g. risk ratio, mean difference) used in the synthesis or presentation of results. | Page 8, line 186 |
| Synthesis methods | 13a | Describe the processes used to decide which studies were eligible for each synthesis (e.g. tabulating the study intervention characteristics and comparing against the planned groups for each synthesis (item #5)). | Page 8, line 181 |
|  | 13b | Describe any methods required to prepare the data for presentation or synthesis, such as handling of missing summary statistics, or data conversions. | N/A |
|  | 13c | Describe any methods used to tabulate or visually display results of individual studies and syntheses. | Page 8, line 182 |
|  | 13d | Describe any methods used to synthesize results and provide a rationale for the choice(s). If meta-analysis was performed, describe the model(s), method(s) to identify the presence and extent of statistical heterogeneity, and software package(s) used. | Page 8, line 182 |
|  | 13e | Describe any methods used to explore possible causes of heterogeneity among study results (e.g. subgroup analysis, meta-regression). | N/A |
|  | 13f | Describe any sensitivity analyses conducted to assess robustness of the synthesized results. | N/A |
| Reporting bias assessment | 14 | Describe any methods used to assess risk of bias due to missing results in a synthesis (arising from reporting biases). | N/A |
| Certainty assessment | 15 | Describe any methods used to assess certainty (or confidence) in the body of evidence for an outcome. | Page 8, line 189 |
| **RESULTS** | | |  |
| Study selection | 16a | Describe the results of the search and selection process, from the number of records identified in the search to the number of studies included in the review, ideally using a flow diagram. | Page 8, line 194 |
|  | 16b | Cite studies that might appear to meet the inclusion criteria, but which were excluded, and explain why they were excluded. | N/A |
| Study characteristics | 17 | Cite each included study and present its characteristics. | Page 23, line 636 |
| Risk of bias in studies | 18 | Present assessments of risk of bias for each included study. | Page 24, line 642 |
| Results of individual studies | 19 | For all outcomes, present, for each study: (a) summary statistics for each group (where appropriate) and (b) an effect estimate and its precision (e.g. confidence/credible interval), ideally using structured tables or plots. | Page 28, line 653 |
| Results of syntheses | 20a | For each synthesis, briefly summarise the characteristics and risk of bias among contributing studies. | Page 8, line 198 |
|  | 20b | Present results of all statistical syntheses conducted. If meta-analysis was done, present for each the summary estimate and its precision (e.g. confidence/credible interval) and measures of statistical heterogeneity. If comparing groups, describe the direction of the effect. | Page 8, line 206 |
|  | 20c | Present results of all investigations of possible causes of heterogeneity among study results. | N/A |
|  | 20d | Present results of all sensitivity analyses conducted to assess the robustness of the synthesized results. | N/A |
| Reporting biases | 21 | Present assessments of risk of bias due to missing results (arising from reporting biases) for each synthesis assessed. | N/A |
| Certainty of evidence | 22 | Present assessments of certainty (or confidence) in the body of evidence for each outcome assessed. | Page 8, line 224 |
| **DISCUSSION** | | |  |
| Discussion | 23a | Provide a general interpretation of the results in the context of other evidence. | Page 13, line 393 |
|  | 23b | Discuss any limitations of the evidence included in the review. | Page 15, line 438 |
|  | 23c | Discuss any limitations of the review processes used. | N/A |
|  | 23d | Discuss implications of the results for practice, policy, and future research. | Page 13, line 356 |
| **OTHER INFORMATION** | | |  |
| Registration and protocol | 24a | Provide registration information for the review, including register name and registration number, or state that the review was not registered. | Page 3, line 37 |
|  | 24b | Indicate where the review protocol can be accessed, or state that a protocol was not prepared. | N/A |
|  | 24c | Describe and explain any amendments to information provided at registration or in the protocol. | N/A |
| Support | 25 | Describe sources of financial or non-financial support for the review, and the role of the funders or sponsors in the review. | Page 16, line 473 |
| Competing interests | 26 | Declare any competing interests of review authors. | Page 16, line 477 |
| Availability of data, code and other materials | 27 | Report which of the following are publicly available and where they can be found: template data collection forms; data extracted from included studies; data used for all analyses; analytic code; any other materials used in the review. | N/A |

**Section II Searching strategies**

**Searching Strategies (PubMed)**

| **Search number** | **Query** |
| --- | --- |
| **1** | ("Ischemic Stroke"[Mesh]) OR (Stroke, Ischemic) OR (Stroke, Ischemic) OR (Ischaemic Stroke) OR (Ischaemic Strokes) OR (Stroke, Ischaemic) OR (Cryptogenic Ischemic Stroke) OR (Cryptogenic Ischemic Strokes) OR (Ischemic Stroke, Cryptogenic) OR (Stroke, Cryptogenic Ischemic) OR (Cryptogenic Stroke) OR (Cryptogenic Strokes) OR (Stroke, Cryptogenic) OR (Cryptogenic Embolism Stroke) OR (Cryptogenic Embolism Strokes) OR (Embolism Stroke, Cryptogenic) OR (Stroke, Cryptogenic Embolism) OR (Wake-up Stroke) OR (Stroke, Wake-up) OR (Wake up Stroke) OR (Wake-up Strokes) OR (Acute Ischemic Stroke) OR (Acute Ischemic Strokes) OR (Ischemic Stroke, Acute) OR (Stroke, Acute Ischemic) |
| **2** | (Andrographolide) OR (Andrographolide) OR (α-bulnesene) OR (L-652469 ) OR (Cedrol) OR (Manoalide) OR (Scalaradial) OR (Kadsurenone) OR (kadsurin) OR (Puberulins) OR (Sarmentine) OR (Piperine) OR (Pipernonaline) OR (piperlongumine) OR (taiwanamide) OR (Magnolol) OR (Honokio) OR (angeloylgomisin P) OR (gomisin A) OR (tigloylgomisin P) OR (L-artigenin) OR (lappaol A) OR (lappaol C) OR (Magnolin) OR (pinoresinol dimethylether) OR (lirioresinol B) OR (nectandrin A) OR (D-dicentrine) OR (quercetin) OR (Kaempferol) OR (Rutin) OR (Myricetin) OR (praeruptorin A and B) OR (tansymustard flavonoids) OR (Scutellarin) OR (Genistein) OR (epigallocatechin gallate) OR (timosaponin D) OR (panax notoginseng saponins) OR (Ginkgo biloba extract ) OR (Ginkgo leaf extract) OR (Rokan) OR (Tanakan) OR (GBE 761) OR (Gingo biloba extract 761) OR (GBE-761) OR (Tebofortran) OR (Tebokan) OR (Tebonin) OR (EGb 761) OR (GBE 761 ONC) OR (EGb-761) OR (EGb761) OR (Ginkgolides) OR (Ginkgolide) OR (ginkgolide J) OR (ginkgolide B) OR (ginkgolide P) OR (ginkgolide Q) OR (ginkgolide X) OR (ginkgolide L) OR (ginkgolide K) OR (ginkgolide A) OR (ginkgolide M) OR (ginkgolide F) OR (ginkgolide C) |
| **3** | (randomized controlled trial[Title/Abstract]) OR (randomized[Title/Abstract])) OR (placebo[Title/Abstract]) |
| **4** | #1 AND #2 AND #3 AND #4 |

**Searching Strategies (CNKI)**

**SU =( ‘银杏内酯’+‘百裕’+‘银杏二萜内酯’+ ‘银杏内酯 A ’+ ‘银杏内酯 B’ + ‘银杏内酯 C’ + ‘银杏内酯 K’ + ‘银杏内酯 J’ + ‘银杏内酯X ’+‘银杏内酯M ’+ ‘银杏内酯P ’+ ‘银杏内酯Q ’+‘银杏内酯L ’+‘银杏内酯F’+‘穿心莲内酯’ + ‘款冬花素’+’雪松醇’ + ‘manoalide’ + ‘scalaradia’+‘槲皮素’+‘山柰酚’ +‘芦丁’+‘杨梅素’+‘白花前胡甲素’ +‘白花前胡乙素’ +‘葶苈子黄酮’ +‘灯盏乙素’ +‘金雀异黄酮’ + ‘表棓儿茶酸酯’ + ‘知母皂苷 D’ + ‘三七总皂苷’+ ‘人参皂苷’ + ‘褐藻多糖硫酸酯’+‘海风藤酮’+ ‘海风藤素’+‘软毛青霉素’+‘假蒟亭碱’+‘胡椒碱’ +‘荜拔环碱’ +‘荜拔明碱’ +‘树胡椒的甲醇提取物’+‘台湾树脂’+‘厚朴酚’+‘当归酰戈P’ +‘五味子醇甲’+‘巴豆酰戈米辛’+‘牛蒡子苷元’+‘木兰脂素’+‘松脂素二甲醚’+‘里立脂素B二甲醚’+‘甘密脂素 A’ + ‘D-荷包牡丹碱’) AND SU=( ‘脑梗死’+‘脑梗’+‘腔隙性脑梗’+ ‘腔梗’+‘脑栓塞’+‘脑血栓’+‘卒中’+‘中风’+ ‘脑缺血’+‘脑血管病’+ ‘急性缺血性脑中风’ +‘急性缺血性脑卒中’) AND TKA=（‘随机对照’+‘随机’+‘对照’+‘RCT’+‘安慰剂’ ）**

**Searching Strategies (WanFang)**

**主题:(银杏内酯 or 百裕 or 银杏二萜内酯 or 银杏内酯 A or 银杏内酯 B or 银杏内酯 C or 银杏内酯 K or 银杏内酯 J or 银杏内酯X or 银杏内酯M or 银杏内酯P or 银杏内酯Q or 银杏内酯L or 银杏内酯F or 穿心莲内酯 or 款冬花素 or 雪松醇 or manoalide or scalaradia or 槲皮素 or 山柰酚 or 芦丁 or 杨梅素 or 白花前胡甲素 or 白花前胡乙素 or 葶苈子黄酮 or 灯盏乙素 or 金雀异黄酮 or 表棓儿茶酸酯 or 知母皂苷 D or 三七总皂苷 or 人参皂苷 or 褐藻多糖硫酸酯 or 海风藤酮 or 海风藤素 or 软毛青霉素 or 假蒟亭碱 or 胡椒碱 or 荜拔环碱 or 荜拔明碱 or 树胡椒的甲醇提取物 or 台湾树脂 or 厚朴酚 or 当归酰戈P or 五味子醇甲 or 巴豆酰戈米辛 or 牛蒡子苷元 or 木兰脂素 or 松脂素二甲醚 or 里立脂素B二甲醚 or 甘密脂素 A or D-荷包牡丹碱) and 主题:(脑梗死 or 脑梗 or 腔隙性脑梗 or 腔梗 or 脑栓塞 or 脑血栓 or 卒中 or 中风 or 脑缺血 or 脑血管病 or 急性缺血性脑中风 or 急性缺血性脑卒中) and 主题:(随机对照 or 随机 or 对照 or 安慰剂 or RCT)**

**Searching Strategies (VIP Database)**

**(题名或关键词=银杏内酯 OR 题名或关键词=百裕) OR 题名或关键词=银杏二萜内酯) OR 题名或关键词=银杏内酯 A) OR 题名或关键词=银杏内酯 B) OR 题名或关键词=银杏内酯 C) OR 题名或关键词=银杏内酯 K) OR 题名或关键词=银杏内酯 J) OR 题名或关键词=银杏内酯X) OR 题名或关键词=银杏内酯M) OR 题名或关键词=银杏内酯P) OR 题名或关键词=银杏内酯 Q) OR 题名或关键词=银杏内酯L) OR 题名或关键词=银杏内酯F) OR 题名或关键词=穿心莲内酯) OR 题名或关键词=款冬花素) OR 题名或关键词=雪松醇) OR 题名或关键词=manoalide) OR 题名或关键词=scalaradia) OR 题名或关键词=槲皮素) OR 题名或关键词=山柰酚) OR 题名或 关键词=芦丁) OR 题名或关键词=杨梅素) OR 题名或关键词=白花前胡甲素) OR 题名或关键词=白花前胡乙素) OR 题名或关键词=葶苈子黄酮 ) OR 题名或关键词=灯盏乙素) OR 题名或关键词=金雀异黄酮) OR 题名或关键词=表棓儿茶酸酯) OR 题名或关键词=知母皂苷 D) OR 题名或 关键词=三七总皂苷) OR 题名或关键词=人参皂苷) OR 题名或关键词=褐藻多糖硫酸酯) OR 题名或关键词=海风藤酮) OR 题名或关键词=海风藤素) OR 题名或关键词=软毛青霉素) OR 题名或关键词=假蒟亭碱) OR 题名或关键词=胡椒碱) OR 题名或关键词=荜拔环碱) OR 题名或关键词=荜拔明碱) OR 题名或关键词=树胡椒的甲醇提取物) OR 题名或关键词=台湾树脂) OR 题名或关键词=厚朴酚) OR 题名或关键词=当归酰戈 P) OR 题名或关键词=五味子醇甲) OR 题名或关键词=巴豆酰戈米辛) OR 题名或关键词=牛蒡子苷元) OR 题名或关键词=木兰脂素) OR 题名或关键词=松脂素二甲醚) OR 题名或关键词=里立脂素B二甲醚) OR 题名或关键词=甘密脂素 A) OR (题名或关键词=D AND ( NOT 题名或关键词=荷包牡丹碱))) AND (((((((((((题名或关键词=脑梗死 OR 题名或关键词=脑梗) OR 题名或关键词=腔隙性脑梗) OR 题名或关键词=腔梗) OR 题名或关键词=脑栓塞) OR 题名或关键词=脑血栓) OR 题名或关键词=卒中) OR 题名或关键词=中风) OR 题名或关键词=脑缺血) OR 题名或关键词=脑血管病) OR 题名或关键词=急性缺血性脑中风) OR 题名或关键词=急性缺血性脑卒中)) AND ((((摘要=随机对照 OR 摘要=随机) OR 摘要=对照) OR 摘要=安慰剂) OR 摘要=RCT)**

**Searching Strategies (Cochrane)**

| **Search number** | **Query** |
| --- | --- |
| **1** | (Stroke, Ischemic):ab,ti,kw OR (Ischaemic Stroke):ab,ti,kw OR (Ischaemic Strokes):ab,ti,kw OR (Stroke, Ischaemic):ab,ti,kw OR (Cryptogenic Ischemic Stroke):ab,ti,kw OR (Cryptogenic Ischemic Strokes):ab,ti,kw OR (Ischemic Stroke, Cryptogenic):ab,ti,kw OR (Stroke, Cryptogenic Ischemic):ab,ti,kw OR (Cryptogenic Stroke):ab,ti,kw OR (Cryptogenic Strokes):ab,ti,kw OR (Stroke, Cryptogenic):ab,ti,kw OR (Cryptogenic Embolism Stroke):ab,ti,kw OR (Cryptogenic Embolism Strokes):ab,ti,kw OR (Embolism Stroke, Cryptogenic):ab,ti,kw OR (Stroke, Cryptogenic Embolism):ab,ti,kw OR (Wake-up Stroke):ab,ti,kw OR (Stroke, Wake-up):ab,ti,kw OR (Wake up Stroke):ab,ti,kw OR (Wake-up Strokes):ab,ti,kw OR (Acute Ischemic Stroke):ab,ti,kw OR (Acute Ischemic Strokes):ab,ti,kw OR (Ischemic Stroke, Acute):ab,ti,kw OR (Stroke, Acute Ischemic):ab,ti,kw |
| **2** | (Andrographolide):ab,ti,kw OR (α-bulnesene):ab,ti,kw OR (L-652469 ):ab,ti,kw OR (Cedrol):ab,ti,kw OR (Manoalide):ab,ti,kw OR (Scalaradial):ab,ti,kw OR (Kadsurenone):ab,ti,kw OR (kadsurin):ab,ti,kw OR (Puberulins):ab,ti,kw OR (Sarmentine):ab,ti,kw OR (Piperine):ab,ti,kw OR (Pipernonaline):ab,ti,kw OR (piperlongumine):ab,ti,kw OR (taiwanamide):ab,ti,kw OR (Magnolol):ab,ti,kw OR (Honokio):ab,ti,kw OR (angeloylgomisin P):ab,ti,kw OR (gomisin A):ab,ti,kw OR (tigloylgomisin P):ab,ti,kw OR (L-artigenin):ab,ti,kw OR (lappaol A):ab,ti,kw OR (lappaol C):ab,ti,kw OR (Magnolin):ab,ti,kw OR (pinoresinol dimethylether):ab,ti,kw OR (lirioresinol B):ab,ti,kw OR (nectandrin A):ab,ti,kw OR (D-dicentrine):ab,ti,kw OR (quercetin):ab,ti,kw OR (Kaempferol):ab,ti,kw OR (Rutin):ab,ti,kw OR (Myricetin):ab,ti,kw OR (praeruptorin A and B):ab,ti,kw OR (tansymustard flavonoids):ab,ti,kw OR (Scutellarin):ab,ti,kw OR (Genistein):ab,ti,kw OR (epigallocatechin gallate):ab,ti,kw OR (timosaponin D):ab,ti,kw OR (panax notoginseng saponins):ab,ti,kw OR (Ginkgo biloba extract ):ab,ti,kw OR (Ginkgo leaf extract):ab,ti,kw OR (Rokan):ab,ti,kw OR (Tanakan):ab,ti,kw OR (GBE 761):ab,ti,kw OR (Gingo biloba extract 761):ab,ti,kw OR (GBE-761):ab,ti,kw OR (Tebofortran):ab,ti,kw OR (Tebokan):ab,ti,kw OR (Tebonin):ab,ti,kw OR (EGb 761):ab,ti,kw OR (GBE 761 ONC):ab,ti,kw OR (EGb-761):ab,ti,kw OR (EGb761):ab,ti,kw OR (Ginkgolides):ab,ti,kw OR (Ginkgolide):ab,ti,kw OR (ginkgolide J):ab,ti,kw OR (ginkgolide B):ab,ti,kw OR (ginkgolide P):ab,ti,kw OR (ginkgolide Q):ab,ti,kw OR (ginkgolide X):ab,ti,kw OR (ginkgolide L):ab,ti,kw OR (ginkgolide K):ab,ti,kw OR (ginkgolide A):ab,ti,kw OR (ginkgolide M):ab,ti,kw OR (ginkgolide F):ab,ti,kw OR (ginkgolide C):ab,ti,kw |
| **3** | #1 AND #2 |

**Searching Strategies (Embase)**

| **Search number** | **Query** |
| --- | --- |
| **1** | 'stroke, ischemic':ab,ti OR 'ischaemic stroke':ab,ti OR 'ischaemic strokes':ab,ti OR 'stroke, ischaemic':ab,ti OR 'cryptogenic ischemic stroke':ab,ti OR 'cryptogenic ischemic strokes':ab,ti OR 'ischemic stroke, cryptogenic':ab,ti OR 'stroke, cryptogenic ischemic':ab,ti OR 'cryptogenic stroke':ab,ti OR 'cryptogenic strokes':ab,ti OR 'stroke, cryptogenic':ab,ti OR 'cryptogenic embolism stroke':ab,ti OR 'cryptogenic embolism strokes':ab,ti OR 'embolism stroke, cryptogenic':ab,ti OR 'stroke, cryptogenic embolism':ab,ti OR 'wake-up stroke':ab,ti OR 'stroke, wake-up':ab,ti OR 'wake up stroke':ab,ti OR 'wake-up strokes':ab,ti OR 'acute ischemic stroke':ab,ti OR 'acute ischemic strokes':ab,ti OR 'ischemic stroke, acute':ab,ti OR 'stroke, acute ischemic':ab,ti |
| **2** | 'andrographolide':ab,ti OR 'α-bulnesene':ab,ti OR 'l-652469':ab,ti OR 'cedrol':ab,ti OR 'manoalide':ab,ti OR 'scalaradial':ab,ti OR 'kadsurenone':ab,ti OR 'kadsurin':ab,ti OR 'puberulins':ab,ti OR 'sarmentine':ab,ti OR 'piperine':ab,ti OR 'pipernonaline':ab,ti OR 'piperlongumine':ab,ti OR 'taiwanamide':ab,ti OR 'magnolol':ab,ti OR 'honokio':ab,ti OR 'angeloylgomisin p':ab,ti OR 'gomisin a':ab,ti OR 'tigloylgomisin p':ab,ti OR 'l-artigenin':ab,ti OR 'lappaol a':ab,ti OR 'lappaol c':ab,ti OR 'magnolin':ab,ti OR 'pinoresinol dimethylether':ab,ti OR 'lirioresinol b':ab,ti OR 'nectandrin a':ab,ti OR 'd-dicentrine':ab,ti OR 'quercetin':ab,ti OR 'kaempferol':ab,ti OR 'rutin':ab,ti OR 'myricetin':ab,ti OR 'praeruptorin a and b':ab,ti OR 'tansymustard flavonoids':ab,ti OR 'scutellarin':ab,ti OR 'genistein':ab,ti OR 'epigallocatechin gallate':ab,ti OR 'timosaponin d':ab,ti OR 'panax notoginseng saponins':ab,ti OR 'ginkgo biloba extract':ab,ti OR 'ginkgo leaf extract':ab,ti OR 'rokan':ab,ti OR 'tanakan':ab,ti OR 'gbe 761':ab,ti OR 'gingo biloba extract 761':ab,ti OR 'gbe-761':ab,ti OR 'tebofortran':ab,ti OR 'tebokan':ab,ti OR 'tebonin':ab,ti OR 'egb 761':ab,ti OR 'gbe 761 onc':ab,ti OR 'egb-761':ab,ti OR 'egb761':ab,ti OR 'ginkgolides':ab,ti OR 'ginkgolide':ab,ti OR 'ginkgolide j':ab,ti OR 'ginkgolide b':ab,ti OR 'ginkgolide p':ab,ti OR 'ginkgolide q':ab,ti OR 'ginkgolide x':ab,ti OR 'ginkgolide l':ab,ti OR 'ginkgolide k':ab,ti OR 'ginkgolide a':ab,ti OR 'ginkgolide m':ab,ti OR 'ginkgolide f':ab,ti OR 'ginkgolide c':ab,ti |
| **3** | 'randomized controlled trial':ab,ti OR 'randomized':ab,ti OR 'placebo':ab,ti |
| **4** | #1 AND #2 AND #3 |

**Searching Strategies (Medline)**

| **#** | **Query** | **Limiters/Expanders** | **Last Run Via** | **Results** |
| --- | --- | --- | --- | --- |
| s4 | ((AB "randomized controlled trial ") OR (AB "randomized") OR (AB " placebo")) AND (S1 AND S2 AND S3) | Expanders - Apply equivalent subjects Search modes - Boolean/Phrase | Interface - EBSCOhost Research Databases Search Screen - Basic Search Database - MEDLINE Complete;CINAHL Complete | 4 |
| s3 | (AB "randomized controlled trial ") OR (AB "randomized") OR (AB " placebo") | Expanders - Apply equivalent subjects Search modes - Boolean/Phrase | Interface - EBSCOhost Research Databases Search Screen - Basic Search Database - MEDLINE Complete;CINAHL Complete | 908,703 |
| s2 | (TI "Andrographolide") OR (TI "α-bulnesene") OR (TI "L-652469 ") OR (TI "Cedrol") OR (TI "Manoalide") OR (TI "Scalaradial") OR (TI "Kadsurenone") OR (TI "kadsurin") OR (TI "Puberulins") OR (TI "Sarmentine") OR (TI "Piperine") OR (TI "Pipernonaline") OR (TI "piperlongumine") OR (TI "taiwanamide") OR (TI "Magnolol") OR (TI "Honokio") OR (TI "angeloylgomisin P") OR (TI "gomisin A") OR (TI "tigloylgomisin P") OR (TI "L-artigenin") OR (TI "lappaol A") OR (TI "lappaol C") OR (TI "Magnolin") OR (TI "pinoresinol dimethylether") OR (TI "lirioresinol B") OR (TI "nectandrin A") OR (TI "D-dicentrine") OR (TI "quercetin") OR (TI"Kaempferol") OR (TI "Rutin") OR (TI "Myricetin") OR (TI "praeruptorin A and B") OR (TI "tansymustard flavonoids") OR (TI "Scutellarin") OR (TI "Genistein") OR (TI "epigallocatechin gallate") OR (TI "timosaponin D") OR (TI "panax notoginseng saponins") OR (TI "Ginkgo biloba extract ") OR (TI "Ginkgo leaf extract") OR (TI "Rokan") OR (TI "Tanakan") OR (TI "GBE 761") OR (TI "Gingo biloba extract 761") OR (TI "GBE-761") OR (TI "Tebofortran") OR (TI "Tebokan") OR (TI "Tebonin") OR (TI "EGb 761") OR (TI "GBE 761 ONC") OR (TI "EGb 761") OR (TI "EGb761") OR (TI "Ginkgolides") OR (TI "Ginkgolide") OR (TI "ginkgolide J") OR (TI "ginkgolide B") OR (TI "ginkgolide P") OR (TI "ginkgolide Q") OR (TI "ginkgolide X") OR (TI "ginkgolide L") OR (TI "ginkgolide K") OR (TI "ginkgolide A") OR (TI "ginkgolide M") OR (TI "ginkgolide F") OR (TI "ginkgolide C") | Expanders - Apply equivalent subjects Search modes - Boolean/Phrase | nterface - EBSCOhost Research Databases Search Screen - Basic Search Database - MEDLINE Complete;CINAHL Complete | 20,812 |
| s1 | (TI "Stroke, Ischemic") OR (TI "Ischaemic Stroke") OR (TI "Ischaemic Strokes") OR (TI "Stroke, Ischaemic") OR (TI "Cryptogenic Ischemic Stroke") OR (TI "Cryptogenic Ischemic Strokes") OR (TI "Ischemic Stroke, Cryptogenic") OR (TI "Stroke, Cryptogenic Ischemic") OR (TI "Cryptogenic Stroke") OR (TI "Cryptogenic Strokes") OR (TI "Stroke, Cryptogenic") OR (TI "Cryptogenic Embolism Stroke") OR (TI "Cryptogenic Embolism Strokes") OR (TI "Embolism Stroke, Cryptogenic") OR (TI "Stroke, Cryptogenic Embolism") OR (TI "Wake-up Stroke") OR (TI "Stroke, Wake-up") OR (TI "Wake up Stroke") OR (TI "Wake-up Strokes") OR (TI "Acute Ischemic Stroke") OR (TI "Acute Ischemic Strokes") OR (TI "Ischemic Stroke, Acute") OR (TI "Stroke, Acute Ischemic") | Expanders - Apply equivalent subjects Search modes - Boolean/Phrase | Interface - EBSCOhost Research Databases Search Screen - Basic Search Database - MEDLINE Complete;CINAHL Complete | 19,186 |

**Section III Appendix Figure 1. Chemical structure of Platelet-Activating Factor Antagonists of Natural Origin** A) Ginkgolide A, B) Ginkgolide B, C) Ginkgolide C, D) Ginkgolide J, E) Ginkgolide K, F) Ginkgolide M, G) Hesperidin, H) Hydroxysafflor yellow A, and I) Ginsenoside Rd.


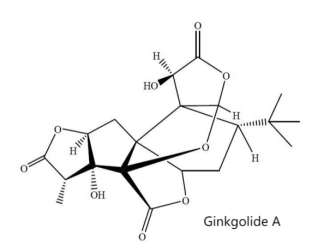

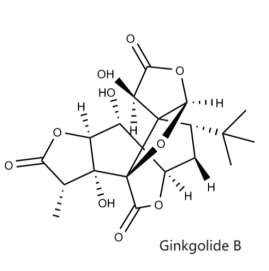


**(A) (B)**


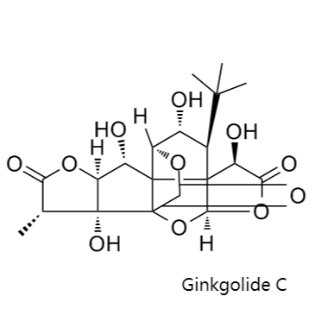

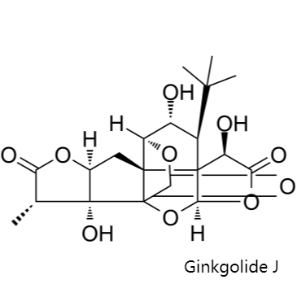


**(C) (D)**


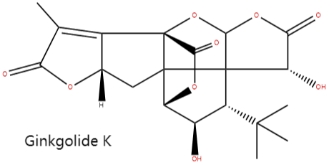

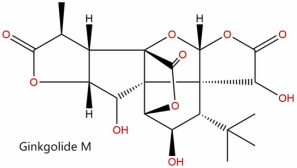


**(E) (F)**


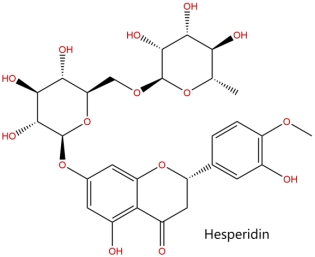

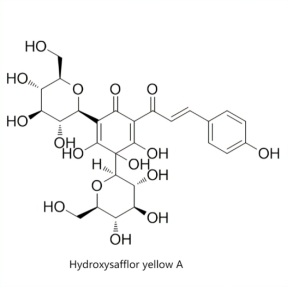


**(G) (H)**


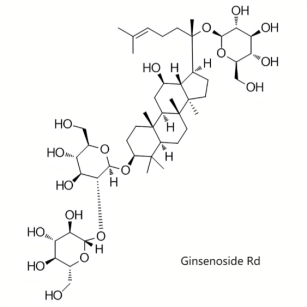


**(I)**

**Section IV Appendix Table 1.Patented formulations,botanical or chemical**

| **Study** | **Formulation** | **Source** | **Species,concentration** | **Quality control reported?(Y/N)** | **Chemical analysis reported?(Y/N)** |
| --- | --- | --- | --- | --- | --- |
| Zhang et al.(2021) | Ginkgolide Injection | Chengdu Baiyu Technology Pharmaceutical Co., Ltd. | - Extracted from Leaf of Ginkgo biloba - Drug Specifications: 2ml per vial, including terpenoids 10mg - Ingredients: bilobalide, Ginkgolide A, Ginkgolide B and Ginkgolide C | Y-Prepared according to Chinese pharmacopeia | Y-HPLC |
| Zhou et al.(2021) | Ginkgolide Injection | Chengdu Baiyu Technology Pharmaceutical Co., Ltd. | - Extracted from Leaf of Ginkgo biloba - Drug Specifications: 2ml per vial, including terpenoids 10mg - Ingredients：bilobalide，Ginkgolide A, Ginkgolide B and Ginkgolide C | Y-Prepared according to Chinese pharmacopeia | Y-HPLC |
| Liu et al.(2018) | Ginkgolide Injection | Chengdu Baiyu Technology Pharmaceutical Co., Ltd. | - Extracted from Leaf of Ginkgo biloba - Drug Specifications: 2ml per vial, including terpenoids 10mg - Ingredients：bilobalide，Ginkgolide A, Ginkgolide B and Ginkgolide C | Y-Prepared according to Chinese pharmacopeia | Y-HPLC |
| Dong et al.(2021) | Ginkgolide Injection | Chengdu Baiyu Technology Pharmaceutical Co., Ltd. | - Extracted from Leaf of Ginkgo biloba - Drug Specifications: 2ml per vial, including terpenoids 10mg - Ingredients：bilobalide，Ginkgolide A, Ginkgolide B and Ginkgolide C | Y-Prepared according to Chinese pharmacopeia | Y-HPLC |
| Su et al.(2018) | Ginkgo Endoterpene Diester Meglumine Injection | Jiangsu Kangyuan Pharmaceutical Co., Ltd. | - Extracted from Leaf of Ginkgo biloba - Drug Specifications: 5ml per vial, including Ginkgo diterpene lactone 25mg - Ingredients：Ginkgolide A, Ginkgolide B and Ginkgolide K | Y-Prepared according to Chinese pharmacopeia | Y-^1^H-NMR(quantitative Nuclear Magnetic Resonance) |
| Feng et al.(2020) | Ginkgo Endoterpene Diester Meglumine Injection | Jiangsu Kangyuan Pharmaceutical Co., Ltd. | - Extracted from Leaf of Ginkgo biloba - Drug Specifications: 5ml per vial, including Ginkgo diterpene lactone 25mg - Ingredients：Ginkgolide A, Ginkgolide B and Ginkgolide K | Y-Prepared according to Chinese pharmacopeia | Y-^1^H-NMR(quantitative Nuclear Magnetic Resonance) |
| Sun et al.(2019) | Ginkgo Endoterpene Diester Meglumine Injection | Jiangsu Kangyuan Pharmaceutical Co., Ltd. | - Extracted from Leaf of Ginkgo biloba - Drug Specifications: 5ml per vial, including Ginkgo diterpene lactone 25mg - Ingredients：Ginkgolide A, Ginkgolide B and Ginkgolide K | Y-Prepared according to Chinese pharmacopeia | Y-^1^H-NMR(quantitative Nuclear Magnetic Resonance) |
| Zheng et al.(2018) | Ginkgo Endoterpene Diester Meglumine Injection | Jiangsu Kangyuan Pharmaceutical Co., Ltd. | - Extracted from Leaf of Ginkgo biloba - Drug Specifications: 5ml per vial, including Ginkgo diterpene lactone 25mg - Ingredients：Ginkgolide A, Ginkgolide B and Ginkgolide K | Y-Prepared according to Chinese pharmacopeia | Y-^1^H-NMR(quantitative Nuclear Magnetic Resonance) |
| Huang et al.(2021) | Ginkgo Endoterpene Diester Meglumine Injection | Jiangsu Kangyuan Pharmaceutical Co., Ltd. | - Extracted from Leaf of Ginkgo biloba - Drug Specifications: 5ml per vial, including Ginkgo diterpene lactone 25mg - Ingredients：Ginkgolide A, Ginkgolide B and Ginkgolide K | Y-Prepared according to Chinese pharmacopeia | Y-^1^H-NMR(quantitative Nuclear Magnetic Resonance) |
| Bao et al.(2019) | Ginkgo Endoterpene Diester Meglumine Injection | Jiangsu Kangyuan Pharmaceutical Co., Ltd. | - Extracted from Leaf of Ginkgo biloba - Drug Specifications: 5ml per vial, including Ginkgo diterpene lactone 25mg - Ingredients：Ginkgolide A, Ginkgolide B and Ginkgolide K | Y-Prepared according to Chinese pharmacopeia | Y-^1^H-NMR(quantitative Nuclear Magnetic Resonance) |
| Wang et al.(2017) | Ginkgo Endoterpene Diester Meglumine Injection | Jiangsu Kangyuan Pharmaceutical Co., Ltd. | - Extracted from Leaf of Ginkgo biloba - Drug Specifications: 5ml per vial, including Ginkgo diterpene lactone 25mg - Ingredients：Ginkgolide A, Ginkgolide B and Ginkgolide K | Y-Prepared according to Chinese pharmacopeia | Y-^1^H-NMR(quantitative Nuclear Magnetic Resonance) |
| Ren et al.(2020) | Ginkgo Bilobate Dropping Pill | Shanxi Qianhui Pharmaceutical Co., Ltd. | - Extracted from Leaf of Ginkgo biloba - Drug Specifications: each pill contains ginkgo ketone ester 5mg - Ingredients: bilobalide, Ginkgolide A, Ginkgolide B , Ginkgolide C, total flavonoids | Y-Prepared according to Chinese pharmacopeia | Y-HPLC-ELSD |
| Qin et al.(2019) | Hesperidin | NM | - Extracted from the Citrus sinesis peel | Y-Prepared according to Chinese pharmacopeia | Y-HPLC |
| Liu et al.(2020) | Ginsenoside Rd Injection | Guangdong Taihe biological pharmaceutical Co., Ltd. | - Extracted from panax ginseng - Drug Specifications: 10mg per vial | Y-Prepared according to Chinese pharmacopeia | Y-HPLC |
| Hu et al.(2020) | Hydroxysafﬂor Yellow A | Guangzhou Youcare Biopharmaceutical Co., Ltd. | - Extracted from safflower carthamus - Drug Specifications: 20mg per vial | Y-Prepared according to Chinese pharmacopeia | Y-HPLC |

**Section V Appendix Figure 2.** Forest plot of Ginkgo Endoterpene Diester Meglumine combined with conventional medicine on mRS.


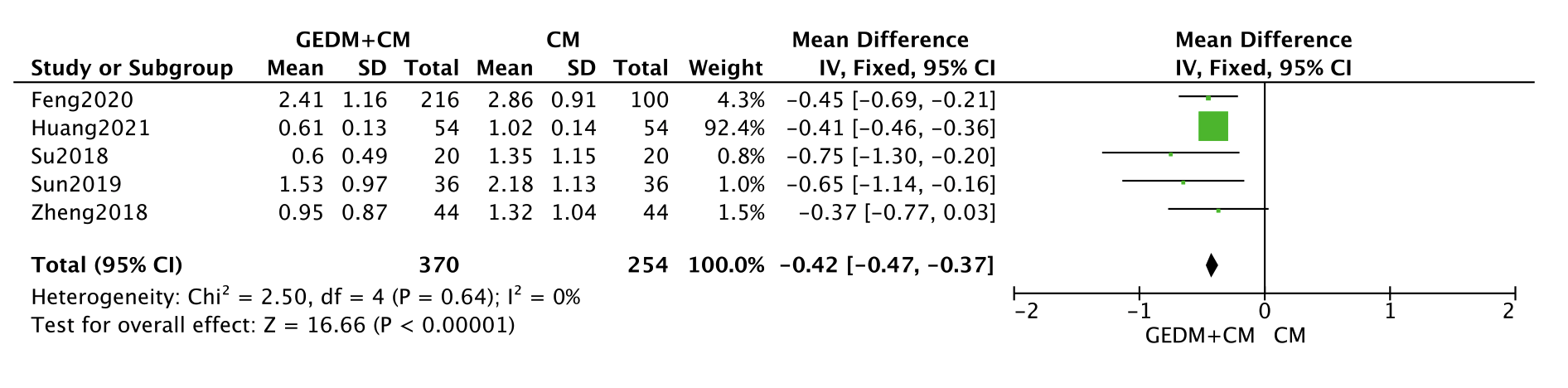


**Section V Appendix Figure 3.** Forest plot of Ginkgo Endoterpene Diester Meglumine combined with conventional medicine on NIHSS.


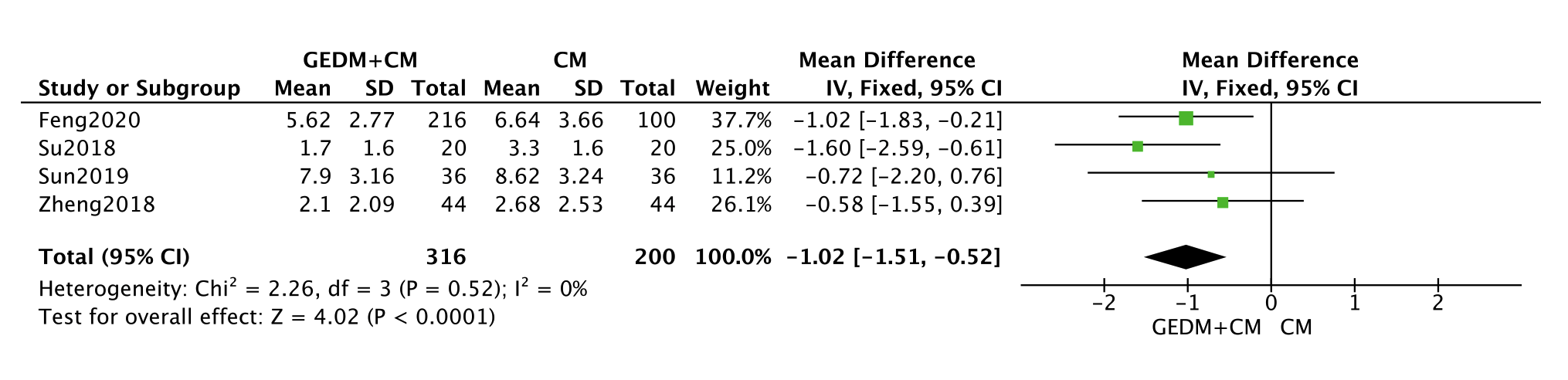


**Section V Appendix Figure 4.** Forest plot of Ginkgo Endoterpene Diester Meglumine combined with edaravone on mRS.


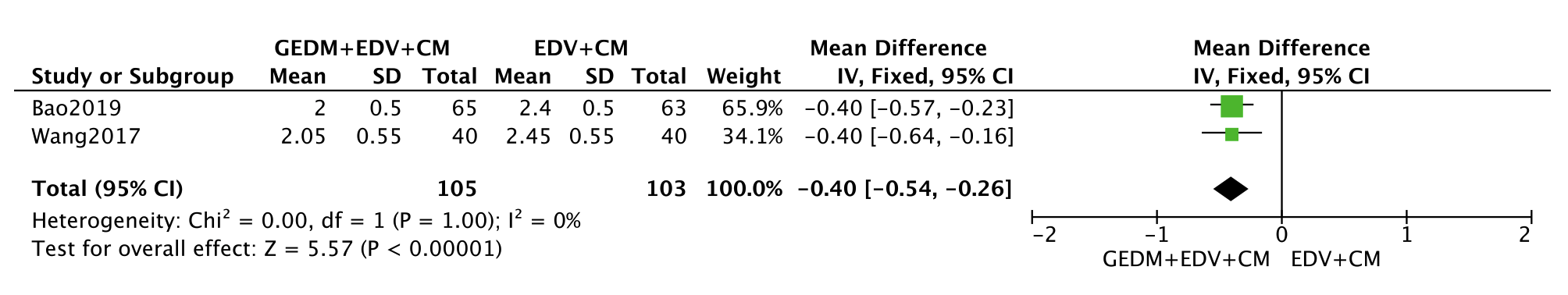


**Section V Appendix Figure 5.** Forest plot of Ginkgo Endoterpene Diester Meglumine combined with edaravone on NIHSS.


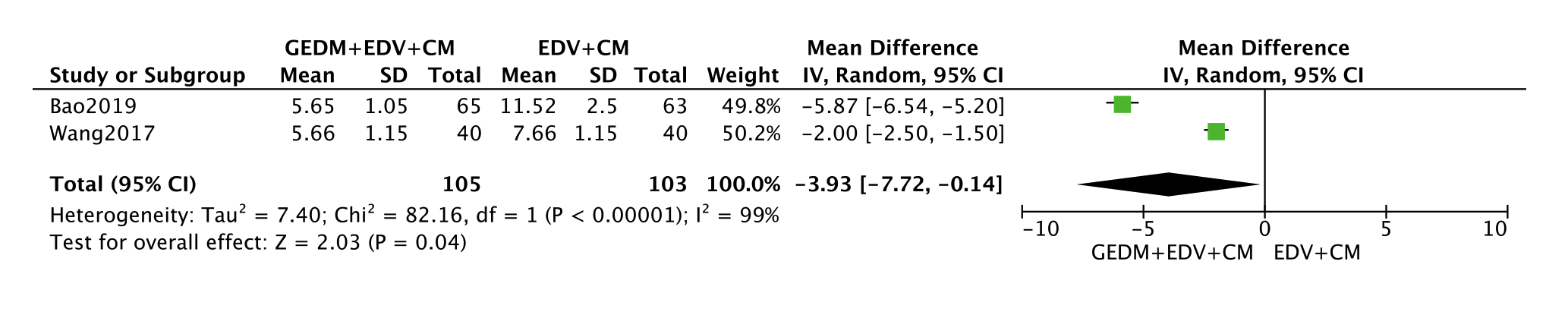


**Section V Appendix Figure 6.** Forest plot of Ginkgo Endoterpene Diester Meglumine combined with other therapies for AIS on mRS (different types of invention).


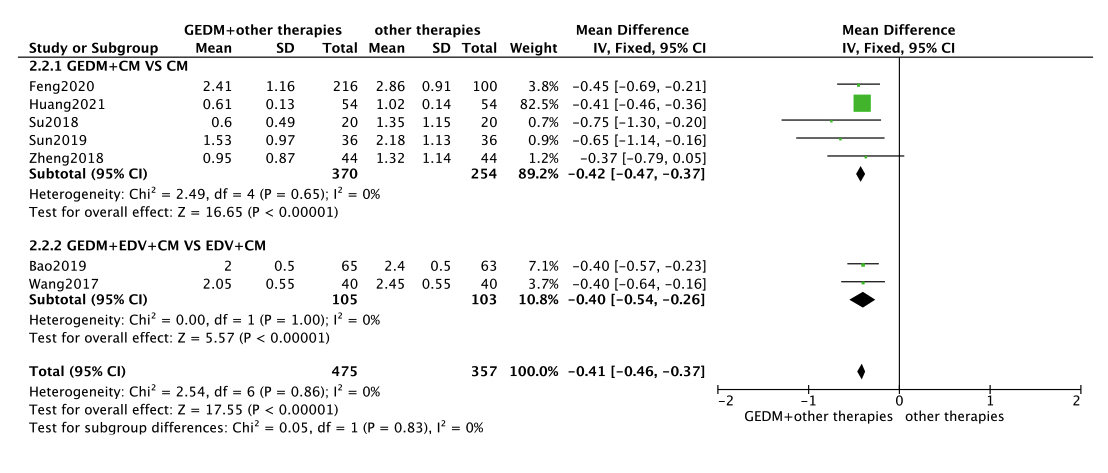


**Section V Appendix Figure 7.** Forest plot of Ginkgo Endoterpene Diester Meglumine combined with other therapies for AIS on NIHSS (different types of invention).


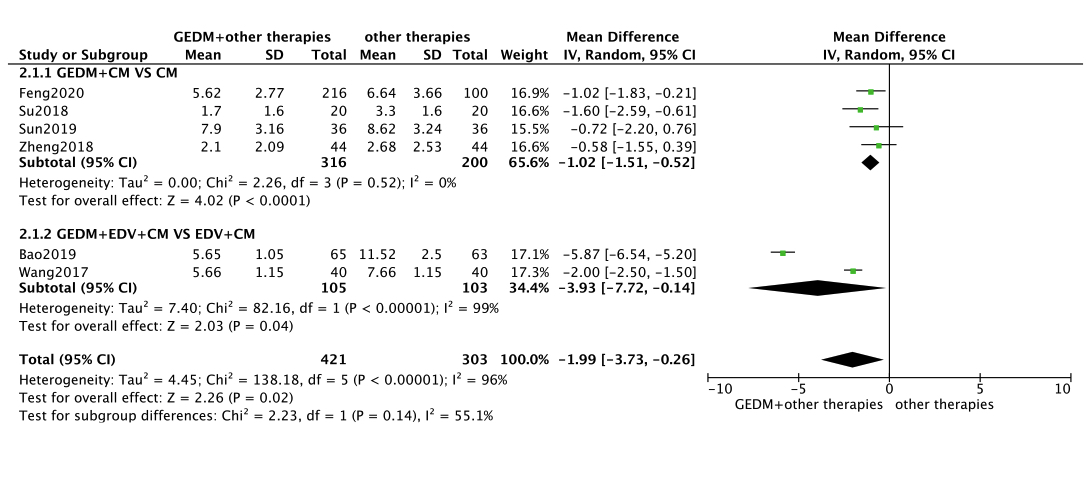


**Section V Appendix Figure 8.** Funnel plot of Ginkgo Endoterpene Diester Meglumine combined with other therapies for AIS on mRS.


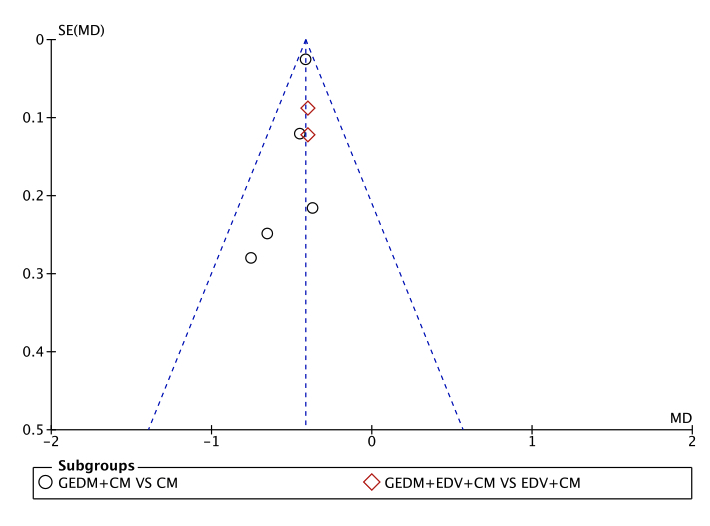


**Section VI Editorial Certificate.**


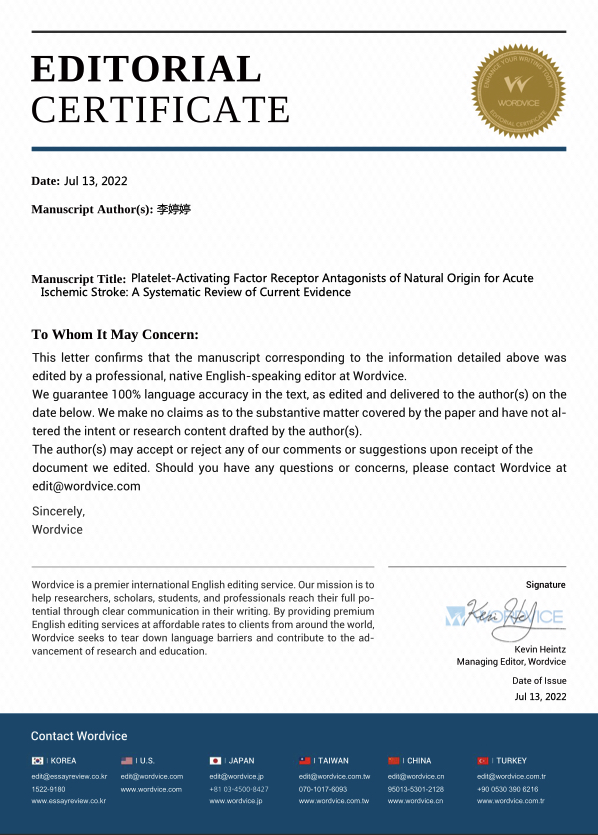


**Section VII Appendix Figure 9.** Platelet-Activating Factor Receptor Antagonists mechanism of action.


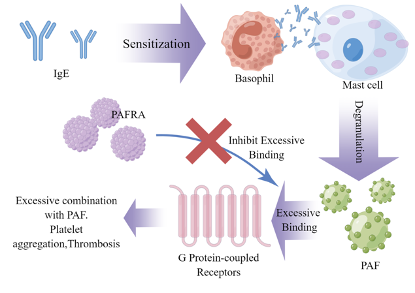

Supplement: Supplementary file 1 [file Table1.doc]
